# *MaTAR25* lncRNA regulates the *Tensin1* gene to impact breast cancer progression

**DOI:** 10.1038/s41467-020-20207-y

**Published:** 2020-12-22

**Authors:** Kung-Chi Chang, Sarah D. Diermeier, Allen T. Yu, Lily D. Brine, Suzanne Russo, Sonam Bhatia, Habeeb Alsudani, Karen Kostroff, Tawfiqul Bhuiya, Edi Brogi, Darryl J. Pappin, C. Frank Bennett, Frank Rigo, David L. Spector

**Affiliations:** 1 Cold Spring Harbor Laboratory, Cold Spring Harbor, NY 11724 New York, USA; 2 Molecular and Cellular Biology Program, Stony Brook University, Stony Brook, NY 11794 USA; 3 Genetics Program, Stony Brook University, Stony Brook, NY 11794 USA; 4 Department of Surgical Oncology, Northwell Health, Lake Success, NY 11042 USA; 5 Department of Pathology, Northwell Health, Lake Success, NY 11042 USA; 6 Memorial Sloan Kettering Cancer Center, New York, NY 10065 USA; 7 Ionis Pharmaceuticals, Carlsbad, CA 92010 USA; 8 Present Address: Department of Biochemistry, University of Otago, Dunedin, 9016 New Zealand

**Keywords:** Breast cancer, Cell biology, Gene regulation, Long non-coding RNAs

## Abstract

Misregulation of long non-coding RNA (lncRNA) genes has been linked to a wide variety of cancer types. Here we report on *Mammary Tumor Associated RNA 25* (*MaTAR25*), a nuclear enriched and chromatin associated lncRNA that plays a role in mammary tumor cell proliferation, migration, and invasion, both in vitro and in vivo. *MaTAR25* functions by interacting with purine rich element binding protein B (PURB), and associating with a major downstream target gene *Tensin1* (*Tns1*) to regulate its expression in *trans*. The Tns1 protein product is a critical component of focal adhesions linking signaling between the extracellular matrix and the actin cytoskeleton. Knockout of *MaTAR25* results in down-regulation of *Tns1* leading to a reorganization of the actin cytoskeleton, and a reduction of focal adhesions and microvilli. We identify *LINC01271 as* the human ortholog of *MaTAR25*, and importantly, increased expression of *LINC01271* is associated with poor patient prognosis and metastasis. Our findings demonstrate that *LINC01271* represents a potential therapeutic target to alter breast cancer progression.

## Abstract (web summary)

A group of long non-coding RNAs (lncRNAs), *Mammary Tumor Associated RNAs* *1-30* (*MaTARs* *1-30*), are differentially expressed between mammary tumor cells and normal mammary epithelial cells. Here the authors report that *MaTAR25* plays a role in breast cancer cell proliferation, migration and invasion by regulating the expression of the *Tensin1* gene in *trans*.

## Introduction

Breast cancer is the most common cancer among women in the United States and worldwide, with an estimated 276,480 new cases of invasive disease in women in the United States in 2020^1^. Although overall breast cancer mortality has been decreasing over the past two decades, it is still the second leading cause of cancer deaths in American women. According to the National Cancer Institute, there will be an estimated 42,170 deaths due to this disease in 2020, accounting for 15% of all cancer deaths among women. Breast tumors can be classified into multiple subtypes based on histological evaluation and the most frequent type of breast tumors are ductal carcinomas, which affect the milk ducts of the breast. Ductal carcinomas can further be separated into two groups: non-invasive ductal carcinoma in situ (DCIS), and invasive ductal carcinoma (IDC) which accounts for 75% of all breast cancers^2,3^. Breast cancer is recognized as a heterogeneous disease and molecular classification of invasive breast carcinomas can stratify tumors into informative subtypes and provide key prognostic signatures. In addition to traditional pathological characterization and immunohistochemistry (IHC) to examine protein levels of markers such as estrogen receptor (ER), progesterone receptor (PR), and epidermal growth factor receptor-2 (HER2), additional studies evaluating genomic rearrangements and molecular expression profiles of breast cancers have provided further genetic insights to better understand the disease^4–6^. These approaches have identified six major molecular subtypes of breast cancer (luminal A, luminal B, HER2-enriched, triple-negative/basal-like, normal breast-like, and claudin-low)^7^, each displaying different phenotypic and molecular features and which have distinct clinical outcomes.

In recent years, large scale genome-wide studies indicated that thousands of RNAs can be transcribed from the human and mouse genomes that lack protein-coding capacity^8–10^. In particular, long non-coding RNAs (lncRNAs) with a length ≥200 nucleotides have been suggested to play key roles in a diverse range of biological processes^11–13^. Most lncRNAs are capped, spliced, and polyadenylated^8^. In addition, many lncRNAs are expressed in a tissue-specific and/or cell-type-specific manner and are involved in various gene-regulatory pathways^14,15^. Furthermore, misregulation of lncRNA expression has been linked to various diseases such as neuromuscular diseases, developmental disorders, neurodegenerative diseases, and cancers^16–20^. Several lncRNAs have been implicated as regulatory molecules in breast cancer progression and metastasis through different mechanisms^21,22^. For example, the *HOX* antisense intergenic RNA (*HOTAIR*) is overexpressed in primary breast tumors and related to cancer progression and metastasis. *HOTAIR* can change the localization pattern of Polycomb repressive complex 2 (PRC2) and histone methylation to regulate gene expression in breast carcinoma cells^23^. Recent findings suggest that the lncRNA breast cancer anti-estrogen resistance 4 (*BCAR4*)^24^ can control GLI family zinc finger 2 (GLI2) gene expression to promote cancer cell migration by interacting with Smad nuclear interacting protein 1 (SNIP1) and serine/threonine-protein phosphatase 1 regulatory subunit 10 (PNUTS). Targeting *BCAR4* by locked nucleic acids (LNA) in mouse models significantly affects cancer cell invasion and reduces lung metastases^25^. Genetic knockout or ASO-mediated knockdown of *Metastasis Associated Lung Adenocarcinoma Transcript 1 (Malat1)* was shown to result in the differentiation of primary mammary tumors and a significant reduction in metastasis^26,27^. In addition to transcriptional regulation, lncRNAs can have other regulatory roles. For example, the lncRNA *PVT1* has been shown to regulate Myc protein stability in breast cancer cells, resulting in cancer cell progression^28^, and the lncRNA *NKILA* can interact with and stabilize the NF-κB/IκB complex and inhibit breast cancer metastasis^29^. However, for the majority of lncRNAs, the exact function and molecular mechanism of action in breast cancers still await detailed characterization. Previously, we performed RNA sequencing (RNA-seq) to identify differentially expressed lncRNAs between mammary tumor cells and normal mammary epithelial cells. From this screen, we identified 30 previously uncharacterized lncRNAs as *Mammary* *Tumor* *Associated* *RNAs* *1-30* (*MaTARs 1–30*)^30^.

Here, we examined the role of *MaTAR25* in mammary tumor progression and metastasis. We found that genetic knockout (KO) of *MaTAR25* in highly aggressive 4T1 triple-negative (ER−, PR−, HER2−) mammary carcinoma cells results in a reduction in cell proliferation, migration, and invasion. KO cells transplanted into the mammary fat pad of BALB/c mice results in a significant decrease in tumor growth as compared to 4T1 control cells. Further, tail-vein injection of luciferase-labeled *MaTAR25* KO cells showed reduced homing to the lungs and a significant decrease in metastatic nodules. In a complementary study, an antisense oligonucleotide (ASO)-mediated knockdown (KD) of *MaTAR25* in the MMTV-Neu-NDL mouse model results in a significant decrease in tumor growth and a reduction in lung metastases. Analysis of the molecular function of *MaTAR25* indicates that it regulates the *Tns1* gene at the transcriptional level. Loss of *MaTAR25* results in a reduction of *Tns1* at the RNA and protein levels and a subsequent reorganization of the actin cytoskeleton and a reduction in focal adhesions and microvilli. Together, our data reveal *MaTAR25*, and its identified human ortholog *LINC01271*, as an exciting therapeutic candidate whose expression can be altered to impede breast cancer progression and metastasis.

## Results

### Characterization of *MaTAR25*, a nuclear-enriched lncRNA

**Fig. 1:**
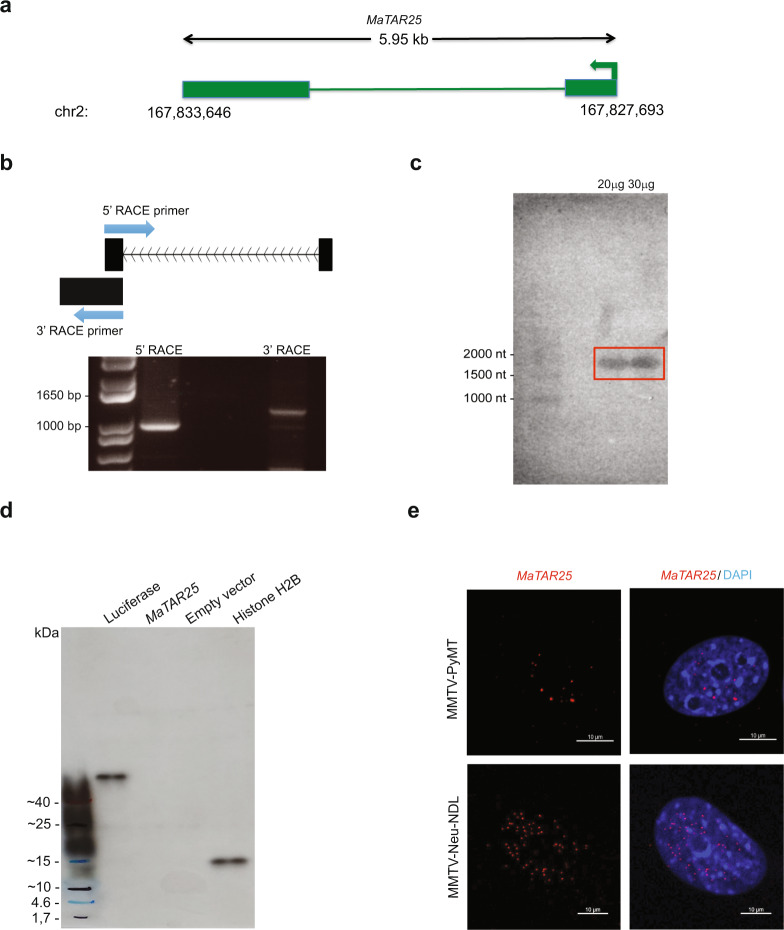
Characterization of *MammaryTumor* *AssociatedRNA25* (*MaTAR25*). **a** Representation of the *MaTAR25* gene locus. *MaTAR25* is an intergenic lncRNA gene located on mouse chromosome 2, and the *MaTAR25* RNA transcript contains 2 exons and a poly (A) tail. **b** 5′ and 3′ rapid amplification of cDNA ends (RACE) was performed to identify the full-length *MaTAR25* transcript. Three independent experiments were performed, and a representative gel image is shown. **c** The full-length *MaTAR25* transcript was confirmed by northern blot analysis to be ~2000 nt. In total, 20 μg or 30 μg of total RNA samples extracted from MMTV-PyMT primary cells were electrophoresed on a 1% agarose gel and probed. Two independent experiments were performed, and a representative gel image is shown. **d** In vitro transcription and translation reactions were performed to confirm that *MaTAR25* does not produce a peptide. The reaction products were loaded on a 4–20% gradient SDS-PAGE gel, and the signals were detected by HRP-conjugated streptavidin. Luciferase control DNA and *Xenopus laevis* Histone H2B (HISTH2B) expressing plasmids were used as positive controls and empty vector as a negative control. Three independent experiments were performed, and a representative gel image is shown. **e** Representative smRNA-FISH images showing localization of *MaTAR25* RNA transcripts (red) within nuclei of MMTV-PyMT and MMTV-Neu-NDL primary cells. Scale bars are 10 μm.

We previously performed an RNA-seq screen to identify lncRNAs overexpressed in mammary tumors vs normal mammary epithelial cells as a means to identify potential candidates involved in mammary cancer progression and to explore their potential as therapeutic targets or key biomarkers in human breast cancer^30^. Among those lncRNA genes identified, the *MaTAR25* gene on mouse chromosome 2 was originally annotated as 1200007C13Rik and it encodes a single transcript containing two exons (Fig. 1a). *MaTAR25* is overexpressed in mammary tumors in the MMTV-Neu-NDL (HER2 subtype) model compared to normal mammary epithelial cells and it is also upregulated in luminal and triple-negative subtypes of mammary cancer^30^. Analysis of ENCODE and FANTOM5 RNA-seq data has shown there is little to no expression of *MaTAR25* in normal mouse tissues compared to MMTV-Neu-NDL tumor cells^30,31^ (Supplementary Fig. 1a). The full-length *MaTAR25* transcript was determined to be 1978 nucleotides by 5′ and 3′ rapid amplification of cDNA ends (RACE) and Sanger sequencing (Fig. 1b), which was further confirmed by northern blot analysis (Fig. 1c).

According to three independent computational coding potential prediction programs, the *MaTAR25* RNA transcript has very low protein-coding potential and is suggested to be a noncoding RNA (Supplementary Fig. 1b–d). However, there is one predicted open reading frame (ORF) with the potential to generate a 123 amino acid peptide (~13 kDa). In order to assess whether a peptide is encoded by the *MaTAR25* transcript, we performed in vitro transcription and translation. Compared to a luciferase DNA control (expected size 61 kDa) and a *Xenopus laevis* Histone H2B (HISTH2B) expressing plasmid control (expected size 14 kDa), there was no detectable peptide generated from a plasmid that contained the *MaTAR25* sequence (Fig. 1d). Together, these computational and experimental results confirm that *MaTAR25* does not make a peptide, and thus is a bona fide lncRNA.

In order to determine the localization and abundance of *MaTAR25*, we performed single-molecule RNA fluorescence in situ hybridization (smRNA-FISH) to detect *MaTAR25* RNA transcripts within MMTV-PyMT (luminal B) and MMTV-Neu-NDL (HER2/neu+) primary mammary tumor cells. The majority of *MaTAR25* transcripts were detected in cell nuclei (Fig. 1e), and each nucleus contained ~10–15 transcript foci. Thus, *MaTAR25* is a nuclear-enriched lncRNA with a potential role in the regulation of gene expression in mammary cancer cells.

### *MaTAR25* knockout decreases 4T1 cell viability/migration/invasion

**Fig. 2:**
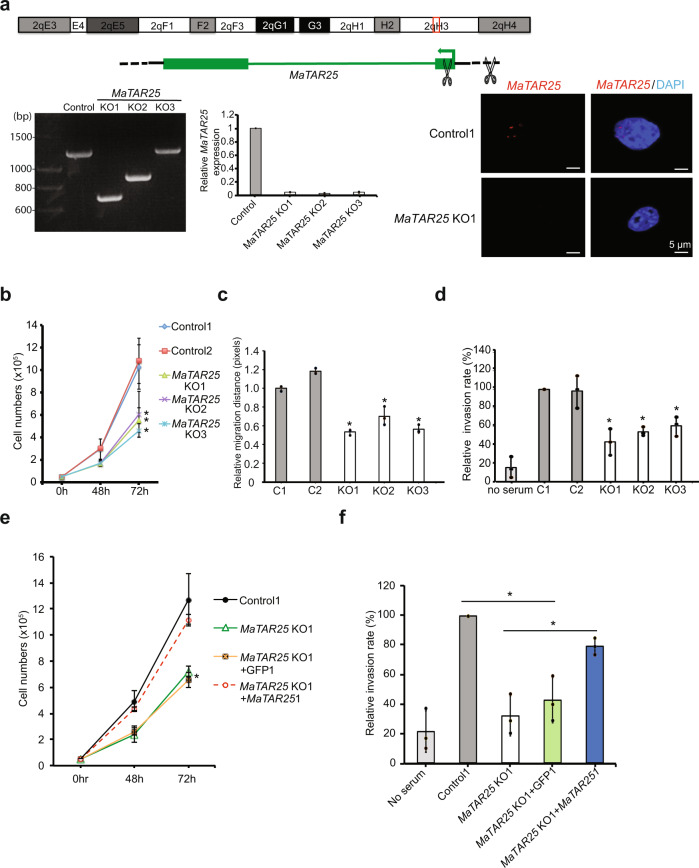
*MaTAR25* knockout affects 4T1 cell viability, migration, and invasion in vitro; all of which can be rescued by ectopic expression of *MaTAR25* in knockout cells. **a** CRISPR/Cas9 was used to generate *MaTAR25* KO clones in 4T1 cells. Pairs of sgRNAs were introduced targeting upstream and downstream of the transcription start site of *MaTAR25*, resulting in 390–620 bp genomic deletions over this region, and a *Renilla* Luciferase sgRNA was used as a negative control. Knockout clones were selected by genomic PCR and Sanger sequencing for homozygous genomic deletion. Control clones were selected by qRT-PCR for expressing similar levels of *MaTAR25* as parental 4T1 cells. qRT-PCR (*n* = 2), and representative images of single smRNA-FISH (red: *MaTAR25*) are shown to confirm *MaTAR25* KO. Scale bars are 5 μm. **b** 4T1 cells were seeded at the same cell density in 12-well tissue culture plates at day 0, and cell counting was performed at different time points. The mean cell numbers of three independent replicates of 4T1 control groups and *MaTAR25* KO groups are shown ± SD (*n* = 3). *P < 0.05 (paired Student’s *t* test; two-tailed). **c** Live-cell tracking was performed over time to examine cell migration. Images were collected every 5 min for a total of 8 h and analyzed by CellTracker image-processing software. The mean relative migration distance (μm) of 4T1 control groups and *MaTAR25* KO groups is shown ± SD (*n* = 3 independent replicates). *P < 0.05 (paired Student’s *t* test; two-tailed). **d** Twenty-four-well Boyden chamber invasion assay (24 h). The mean relative cell invasion of three independent replicates of 4T1 control groups and *MaTAR25* KO groups is shown ± SD (*n* = 3). *P < 0.05 (paired Student’s *t* test; two-tailed). **e** Selected clones with ectopic expression of *MaTAR25* or GFP were used as positive and negative controls to assess rescue in a cell viability assay. Data are presented as mean values ± SD (*n* = 3). *P < 0.05 (paired Student’s *t* test; two-tailed). **f** Cell invasion assay. The mean cell numbers and mean relative cell invasion of three independent replicates of 4T1 control1, *MaTAR25* KO1, *MaTAR25* KO1 with GFP expression, and *MaTAR25* KO1 with *MaTAR25* ectopic expression is shown ± SD (*n* = 3). *P < 0.05 (paired Student’s *t* test; two-tailed).

To assess the functional role of *MaTAR25*, we proceeded to genetically KO *MaTAR25* in highly aggressive 4T1 triple-negative (ER−, PR−, HER2−) mammary carcinoma cells using CRISPR/Cas9. We designed gRNA pairs targeting various regions upstream and downstream of the transcription start site (TSS) of *MaTAR25* to create a genomic deletion (Fig. 2a and Supplementary Fig. 2a). *MaTAR25* knockout clones were single-cell sorted and selected by Sanger sequencing, qRT-PCR, as well as single-molecule RNA-FISH (Fig. 2a and Supplementary Fig. 2b).

After selecting several *MaTAR25* KO clones, we evaluated them for alterations in cell viability, migration, and invasion as compared to control 4T1 cells. *MaTAR25* KO cells exhibited a significant decrease of 50% in cell viability as compared to 4T1 control cells (Fig. 2b). To further investigate this phenotype, we performed BrdU labeling and FACS analysis, which demonstrated a twofold increase in G2 cells, suggesting that the decreased proliferation phenotype is most likely the result of a lengthened G2 phase (Supplementary Fig. 2c). As cell migration and invasion are critical processes associated with metastasis, we were interested in determining whether *MaTAR25* loss might play a role in these events. We used a live-cell tracking assay to assess cell migration and we found a 40% reduction in cell motility upon loss of *MaTAR25* (Fig. 2c, Supplementary Fig. 2d, and Supplementary Movies 1, 2). A wound-healing assay also corroborated the observed difference in cell migration between 4T1 control and *MaTAR25* KO cells (Supplementary Fig. 2e). Finally, we used a Boyden chamber invasion assay and found that loss of *MaTAR25* resulted in a 45% reduction in invasion ability as compared to 4T1 control cells (Fig. 2d).

In order to exclude the possibility that the phenotypes observed in *MaTAR25* KO cells were caused by disturbing chromatin structure rather than specific loss of the *MaTAR25* transcript, we generated single-cell ectopic overexpression clones of *MaTAR25* in 4T1 *MaTAR25* KO cells. Ectopic expression of *MaTAR25* rescued both the cell proliferation and invasion phenotypes (Fig. 2e, f), indicating that *MaTAR25* RNA plays an important role in these processes in situ, and likely exhibits its effect in *trans*. Hence, *MaTAR25* appears to be an important lncRNA impacting mammary tumor cell growth and critical aspects of metastasis. To further explore *MaTAR25’s* downstream targets, we performed RNA-seq to identify differentially expressed genes by comparing *MaTAR25* KO cells with 4T1 control cells (Supplementary Data 1). Pathway analysis of the differentially expressed genes by Kyoto Encyclopedia of Genes and Genomes (KEGG) and Gene Set Enrichment Analysis (GSEA) both revealed alteration in cell cycle and DNA-related processes, both related to the observed phenotypes we observed in *MaTAR25* KO cells (Supplementary Fig. 2f). The expression levels of three neighboring protein-coding genes (*Snai1, Cebpb,* and *Ptpn1*) were not consistently altered in *MaTAR25* KO cells compared to 4T1 control cells (Supplementary Fig. 2g). In addition, the expression levels of these three neighboring protein-coding genes were not significantly changed upon *MaTAR25*-specific antisense oligonucleotide (ASO)-mediated knockdown in 4T1 and cNeu (MMTV-Neu-NDL) cells consistent with the role of *MaTAR25* functioning in *trans* (Supplementary Fig. 2h).

### *MaTAR25* knockout decreases tumor progression/metastasis in vivo

**Fig. 3:**
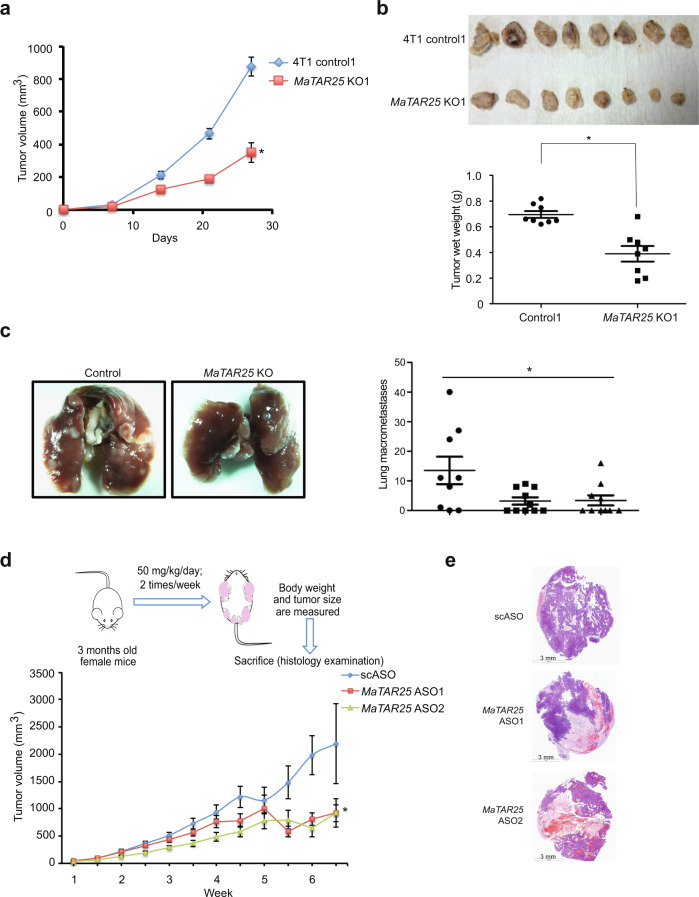
*MaTAR25* knockout impairs tumor growth and metastasis in vivo. **a** 4T1 control or *MaTAR25* KO cells were injected orthotopically into the mammary fat pad of female BALB/c mice. Primary tumors were measured every week over a period of 4 weeks, and the mean tumor volume of eight mice per group is shown ± SE. *P < 0.05 (paired Student’s *t* test; two-tailed). **b** Ten mice were sacrificed, and tumors were collected at day 28 to compare the tumor growth rate between the control group and *MaTAR25* KO groups. Tumors derived from *MaTAR25* KO cells showed a 56% reduction in tumor growth. The mean tumor wet weight is shown ± SE. *P < 0.05 (paired Student’s *t* test; two-tailed). **c** Female BALB/c mice were injected into the tail vein with 4T1 control1 or *MaTAR25* KO cells. Mice were monitored every week and sacrificed at day 21. Mouse lungs were collected and imaged (left panel), and lung metastatic nodules were counted to compare the metastatic ability between the control group and *MaTAR25* KO group (right panel). Mice injected with *MaTAR25* KO cells exhibited a 62% reduction in lung metastatic nodules. Data are presented as mean values shown ± SD. *P < 0.05 (paired Student’s *t* test; two-tailed). **d** Schematic showing the approach for ASO-mediated knockdown of *MaTAR25* in MMTV-Neu-NDL mice. Two independent *MaTAR25* ASOs or a control scASO were used for subcutaneous injection. Primary tumors were measured twice per week, and the mean tumor volume of seven mice per group is shown ± SE. *P < 0.05 (paired Student’s *t* test; two-tailed). **e** Hematoxylin and eosin (H&E)-stained tumor images showing the different histological phenotypes between tumor samples from the scASO-injected group and *MaTAR25* ASO-injected group. More than five tumor samples in each group were examined, and representative images are shown. Scale bars are 3 mm.

In order to further evaluate the functional impact and the therapeutic potential of *MaTAR25* in mammary tumor progression, we performed multiple in vivo studies. Injection of *MaTAR25* 4T1 KO cells into the mammary fat pad of BALB/c mice resulted in a significant 56% decrease in tumor growth at day 28, compared to the 4T1 control injected group (Fig. 3a, b). In addition, we performed tail-vein injection using *MaTAR25* KO cells expressing a luciferase reporter to track cancer cell homing and metastasis to the lungs in BALB/c mice. The in vivo bioluminescence signal in the lungs of mice injected with *MaTAR25* KO cells was reduced (Supplementary Fig. 3a) compared to those injected with 4T1 control cells. On day 21, the number of metastatic nodules in lung samples collected from the *MaTAR25* KO group was also significantly decreased by 62% compared to the 4T1 control group (Fig. 3c).

As a complementary approach to CRISPR/Cas9 KO, we designed a series of *MaTAR25*-specific antisense oligonucleotides (ASOs), (16-mers) comprised of phosphorothioate-modified short S-cEt (S-2′-O-Et-2′,4′-bridged nucleic acid) gapmer chemistry^32–34^. We individually screened multiple ASOs targeting *MaTAR25* to identify the most effective ASOs in terms of KD efficiency by qRT-PCR after 48 h and 72 h of ASO treatment in 4T1 cells. The two most effective *MaTAR25* ASOs achieved a knockdown ranging from 70 to 90% (Supplementary Fig. 1e). When comparing *MaTAR25* ASO-treated cells to mock or scrambled ASO (scASO)-treated 4T1 control cells after 72 h, we found a significant decrease in cell viability using cell counting assays (−45% for ASO1 and −38% for ASO2) (Supplementary Fig. 1f), and similar reduction phenotypes were also observed in cNeu (MMTV-Neu-NDL) cells treated with *MaTAR25* ASOs (−50% for ASO1 and −41% for ASO2) (Supplementary Fig. 1g), consistent with our KO studies indicating that *MaTAR25* has a role in mammary cancer cell proliferation.

Furthermore, to assess the therapeutic potential of reducing the level of *MaTAR25* in vivo, we evaluated the impact of subcutaneous injection of two independent *MaTAR25* ASOs for their in vivo ability to knockdown *MaTAR25* and to impact mammary tumor progression in the MMTV-Neu-NDL mouse model. ASO-mediated knockdown of *MaTAR25* resulted in a 59% decrease in tumor growth compared to the scASO control group (Fig. 3d and Supplementary Fig. 3b). By comparing the hematoxylin and eosin (H&E)-stained tumor sections collected from the *MaTAR25* ASO-injected group with the scASO control group, we observed a strong level of necrosis in the *MaTAR25* ASO-treated mammary tumor samples (Fig. 3e) but not in other non-tumor tissues (Supplementary Fig. 3c). Importantly, mammary tumors from the scASO control group lacked any significant necrotic phenotype (Fig. 3e). We also collected lung samples from each group to examine for the presence of micro-metastases, and the H&E-stained lung sections showed that KD of *MaTAR25* resulted in a 40% incidence rate of micro-metastatic nodules in lungs from ASO1 or ASO2-treated animals as compared to a 76.9% incidence rate for the scASO control group (Supplementary Fig. 3d). Together, our in vitro and in vivo data indicate that *MaTAR25* plays a critical role in promoting mammary tumor progression and metastasis.

### *MaTAR25* is a positive upstream regulator of *Tns1*, a mediator of cell–matrix adhesion and migration

**Fig. 4:**
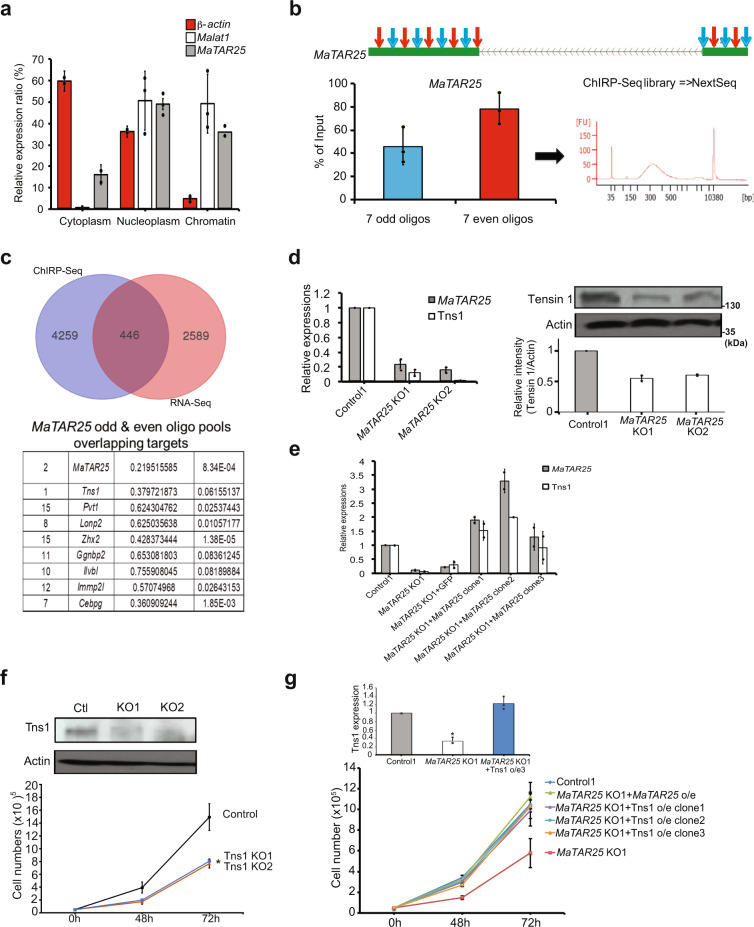
*MaTAR25* is a positive upstream regulator of *Tns1*. **a** Cell fractionation was performed to isolate cytoplasmic, nucleoplasmic, and chromatin-associated RNA. qRT-PCR was used to determine the subcellular localization ratio of *MaTAR25* transcripts. *β-actin* and *Malat1* were used as marker RNAs for quality control. Data are presented as mean values ± SD (*n* = 3 independent experiments). **b** Schematic diagram showing the targeting of biotin-labeled oligonucleotides binding *MaTAR25* transcripts for Chromatin Isolation by RNA Purification (ChIRP)-seq. Odd and even oligo pools (seven oligos in each pool) were used, and qRT-PCR was performed to check the RNA purification efficiency. Data are presented as mean values ± SD (*n* = 3 independent experiments). **c** Venn diagram showing differentially expressed genes in *MaTAR25* KO cells identified from RNA-Seq overlapped with *MaTAR25* ChIRP-seq data. The top candidate genes are listed. Statistics were determined using DESeq2, and a FDR-adjusted *P* value of <0.1 was set as a threshold for statistical significance. **d** Validation of *Tns1* as a *MaTAR25*-targeted gene by qRT-PCR and immunoblotting in 4T1 control and *MaTAR25* KO cells (*n* = 2 independent experiments). **e** The RNA expression level of *Tns1* is rescued upon ectopic expression of *MaTAR25* in *MaTAR25* KO cells as determined by qRT-PCR (*n* = 2 independent experiments). **f** CRISPR/Cas9 targeting was used in 4T1 cells to generate *Tns1* knockout clones. The upper panel shows expression levels of Tns1 in 4T1 control, *Tns1* KO clone1, and *Tns1* KO clone2 by immunoblotting. The lower panel shows the cell counting viability assay result of 4T1 control1, *Tns1* KO1, and *Tns1* KO2. Results are mean ± SD (*n* = 3 independent experiments). *P < 0.05 (paired Student’s *t* test; two-tailed). **g** Ectopic expression of *Tns1* in *MaTAR25* KO cells rescues the cell viability defect. The top panel shows expression levels of Tns1 in 4T1 control1, *MaTAR25* KO1, *MaTAR25* KO1 with *Tns1* ectopic expression clone3 by qRT-PCR. Results are mean ± SD (*n* = 3 independent experiments). *P < 0.05 (paired Student’s *t* test; two-tailed). The bottom panel shows the cell counting viability assay results of 4T1 control1, *MaTAR25* KO1, *MaTAR25* KO1 with *MaTAR25* ectopic expression, and *MaTAR25* KO1 with *Tns1* ectopic expression clone1-3. Results are mean ± SD (*n* = 2 independent experiments).

Next, we were interested in revealing aspects of the molecular mechanism of action of *MaTAR25* in regulating mammary tumor progression. Since we previously identified *MaTAR25* to be highly enriched in cell nuclei by smRNA-FISH, we went on to perform cell fractionation to isolate cytoplasmic and nucleoplasmic lysates as well as chromatin pellets of 4T1 cells to determine the subcellular enrichment of *MaTAR25* by qRT-PCR analysis. Notably, compared to the enrichment of β*-actin* and *Malat1*, we found a significant enrichment of *MaTAR25* in the nucleoplasmic and chromatin fractions (Fig. 4a), indicating that the molecular mechanism of action of *MaTAR25* may be related to transcriptional regulation. To test this hypothesis, we performed Chromatin Isolation by RNA Purification (ChIRP)^35^ to pull down RNA/DNA complexes by using specific biotin-labeled antisense oligonucleotides targeting *MaTAR25* as well as biotin-labeled antisense oligonucleotides targeting housekeeping gene Ppib transcripts as the corresponding control (Fig. 4b). ChIRP-seq identified *MaTAR25* genomic targeting sites (Supplementary Data 2, 3), and revealed that these targets are highly enriched in simple repeats regions and LTRs (log ratio enrichments to input are two- to threefold). According to Hypergeometric Optimization of Motif EnRichment (HOMER) motif analysis, these targets are potential binding sites of the transcription factors ZBTB7C, ZNF354C, Irf3, GATA1, and REL (Supplementary Fig. 4a). Combining the *MaTAR25* KO RNA-seq data and ChIRP-seq results, we found a total of 446 overlapping genes (Fig. 4c and Supplementary Data 4), which could be downstream targets regulated by *MaTAR25*. Among these overlapping genes, the top gene ranked by ChIRP-seq data, just under *MaTAR25* itself, is *Tensin1* (*Tns1*) (Fig. 4c and Supplementary Fig. 4b). The *Tns1* gene encodes for a protein that localizes to focal adhesions and positively regulates cell migration and invasion^36,37^. By qRT-PCR and immunoblot analysis, we found that the RNA and protein levels of *Tns1* are significantly lower in *MaTAR25* KO cells than in 4T1 control cells (Fig. 4d). Additional qRT-PCR results from *MaTAR25* ASO1-mediated KD in 4T1 and cNeu cells also indicated *Tns1* expression levels correlate with *MaTAR25* RNA levels (Supplementary Fig. 4d). Interestingly, ectopic expression of *MaTAR25* in *MaTAR25* KO cells results in a corresponding increase in the level of *Tns1* (Fig. 4e). Hence, we conclude that *Tns1* is a direct downstream target of *MaTAR25* and further confirming that *MaTAR25* imparts its function in *trans*. In order to confirm the ChIRP-seq result and to further investigate how *MaTAR25* might regulate the level of *Tns1*, we next performed double-label DNA FISH to detect the *MaTAR25* (Chr2) and *Tns1* (Chr1) gene loci in cells, and we found no physical interaction between these genomic loci (Supplementary Fig. 4e, upper panels). However, combined *MaTAR25* smRNA-FISH and *Tns1* DNA FISH in the same cells showed that *MaTAR25* RNA was overlapping with at least one *Tns1* allele in 50% of the cells (Supplementary Fig. 4e). In contrast, RNA-FISH to localize *MaTAR26* only showed a 14% overlap between the *MaTAR26* RNA signal and a *Tns1* allele (Supplementary Fig. 4e). This demonstrates the specificity of *MaTAR25* RNA transcripts associating with the *Tns1* gene to regulate its expression. We, therefore, performed CRISPR/Cas9 knockout using gRNAs targeting *Tns1* in 4T1 cells and selected *Tns1* KO clones for in vitro functional assays. We found that the *Tns1* KO cells phenocopied the *MaTAR25* KO cells and exhibited a significant 40% decrease in cell viability (Fig. 4f) and a 30% decrease in cell migration vs control cells (Supplementary Fig. 4f). In addition, ectopic expression of *Tns1* in 4T1 *MaTAR25* KO cells can rescue the cell viability phenotype (Fig. 4g). Interestingly, high expression of *TNS1* is strongly correlated with poor survival of grade 3 breast cancer patients^38^ (Supplementary Fig. 4c). Together, these data indicate that *Tns1* is a critical downstream target of *MaTAR25*.

Since *Tns1* is a key component of focal adhesion complexes and is responsible for cell–cell and cell–matrix interactions as well as cell migration by interacting with actin filaments^39^, we examined the organization of actin filaments, as well as the additional focal adhesion complex components paxillin and vinculin^40^, in 4T1 control and *MaTAR25* KO cells by immunofluorescence (IF) confocal microscopy. Indeed, the F-actin microfilaments are disrupted (Supplementary Fig. 4g), and the distribution of paxillin and vinculin proteins are altered dramatically (Supplementary Fig. 4i) in 4T1 *MaTAR25* KO cells as compared to 4T1 control cells. Interestingly, both ectopic expression of *MaTAR25* or *Tns1* in 4T1 *MaTAR25* KO cells can rescue the actin filament phenotype (Supplementary Fig. 4g), supporting our finding that *Tns1* is a critical downstream target of *MaTAR25* regulating mammary tumor progression. To further evaluate the phenotype of *MaTAR25* KO cells, we used transmission electron microscopy (TEM) (Supplementary Fig. 4h). TEM clearly revealed a dramatic reduction of microvilli over the cell surface of *MaTAR25* KO cells compared to 4T1 control cells, indicating loss of *MaTAR25* expression impacts the actin-bundling process (Supplementary Fig. 4h) as well as microvilli formation in 4T1 cells.

### *MaTAR25* interacts with PURB to carry out its function

**Fig. 5:**
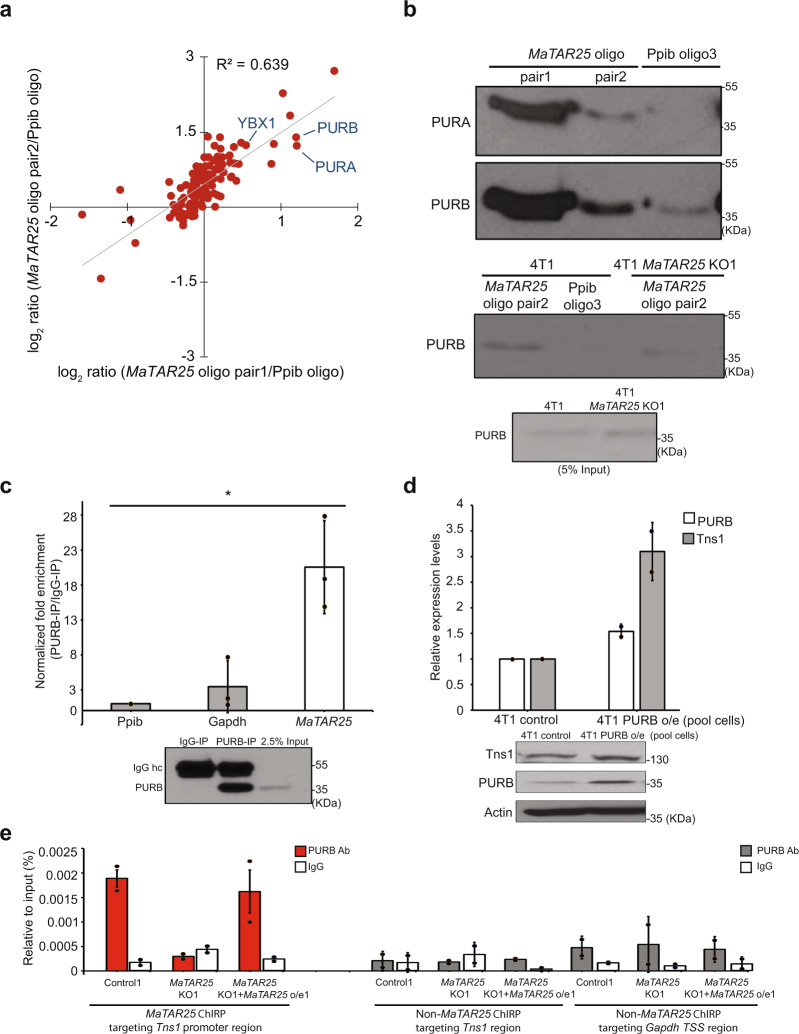
*MaTAR25* interacts with PURB to carry out its function. **a** Scatterplot depicts the fold enrichment of protein candidates from isobaric tags for the relative and absolute quantitation (iTRAQ) analysis comparing two independent oligo pair sets targeting *MaTAR25* RNA transcripts vs Ppib RNA transcripts. **b** Upper: immunoblot analysis of PURA and PURB following pulldown of *MaTAR25* or Ppib from 4T1 cells. Lower: immunoblot analysis of PURB following the pulldown of *MaTAR25* or Ppib from 4T1 cells or 4T1 *MaTAR25* KO cells. More than three different experiments were performed, and representative images are shown. **c** *MaTAR25*, Ppib, and Gapdh transcripts were assessed by qRT-PCR in endogenous PURB, or IgG (negative control) immunoprecipitates from 4T1 cells. Fold enrichment of PURB associated RNA signal over IgG signal is calculated and data are presented as mean values ± SD (*n* = 3 independent experiments). *P < 0.05 (paired Student’s *t* test; two-tailed). Immunoblot analysis of PURB was performed as a control. **d** qRT-PCR analysis and immunoblotting of Tns1 expression in 4T1 cells following ectopic overexpression of PURB. The relative expression levels are shown as mean values ± SD (*n* = 2 independent experiments). **e** ChIP-qPCR analysis of PURB occupancy over the identified *MaTAR25* targeting region and non-targeting region of the *Tns1* DNA locus by ChIRP-seq analysis. ChIP-qPCR was performed in 4T1 control1 cells, 4T1 *MaTAR25* KO1 cells, and upon ectopic expression of *MaTAR25* in *MaTAR25* KO1 cells. Primers for a *MaTAR25* non-targeting region and the Gapdh TSS were used as negative controls. Bar graphs represent the mean ± SD (*n* = 2 independent experiments).

It has been suggested that lncRNAs can interact with transcriptional regulators/co-factors to form ribonucleoprotein (RNP) complexes to regulate the expression of downstream genes in the cell nucleus^41^. To identify *MaTAR25* interacting proteins, we used two different paired sets of biotin-labeled antisense oligonucleotides targeting *MaTAR25* for native RNA antisense oligonucleotide pulldown (RAP) in 4T1 cells followed by qRT-PCR which revealed a 50–60% pull-down efficiency (Supplementary Fig. 5a). Samples were eluted from beads for mass spectrometry isobaric tags for relative and absolute quantitative (MS-iTRAQ) analysis to identify proteins that bind to *MaTAR25*, and Ppib as the corresponding control. We ranked the candidate interactors based on detectable peptides above background in both pair sets of oligonucleotide pull-downs, and selected candidates with at least twofold enrichment compared to corresponding Ppib oligo pulldown (Fig. 5a and Supplementary Data 9). Among the protein candidates, two transcription co-regulators always appeared on the top list between multiple runs. These are purine-rich element-binding protein A (PURA) and purine-rich element-binding protein B (PURB), which can form homodimers or heterodimers in the nucleus^42^. Additionally, one other protein, Y-box protein 1 (YBX1) also on the candidate list, but which did not pass the enrichment criteria, was shown in a previous study to interact with PURA to form a PURB/PURA/YBX1 heterotrimer^43^. To verify our MS result, we first performed immunoblot analysis with PURA and PURB antibodies and we could detect significantly higher enrichment of PURA and PURB in samples of *MaTAR25* oligonucleotide pulldown than in the Ppib oligonucleotide pulldown (Fig. 5b, upper panel). RNA immunoprecipitation (RIP) using PURB antibodies compared to IgG control also revealed the specificity of the *MaTAR25*-PURB interaction (Fig. 5c) but not *MaTAR25*-PURA (Supplementary Fig. 5b). Based on these data, we demonstrate that PURB is the lead protein directly binding to *MaTAR25*. Immunoblot analysis using pulldown samples from 4T1 and 4T1 *MaTAR25* KO cell lysates (Fig. 5b, lower panel) plus pull-down samples from NDL primary cell lysates (Supplementary Fig. 5c) confirmed the specific interaction between *MaTAR25* and PURB. To further confirm the role of PURB in regulating *Tns1*, we manipulated the level of PURB in 4T1 cells either through ectopic overexpression (Fig. 5d) or siRNA-mediated knockdown and demonstrated upregulation or downregulation of *Tns1*, respectively (Supplementary Fig. 5d). The results confirmed that the expression level of *Tns1* is related to the changes in PURB expression level in 4T1 cells, indicating that the *MaTAR25*/PURB RNP complex is essential for the regulation of *Tns1*.

*Tns1* isoform 3 was identified as the major isoform expressed in MMTV-Neu-NDL and 4T1 cells (Supplementary Fig. 5e). Based on our ChIRP-seq result, we were able to go on to identify the promoter region of *Tns1* isoform 3, which contains a very high purine:pyrimidine ratio (3:1) including many potential PURB-binding sequence motifs (GGTGG)^44^, as the main targeting region in the *Tns1* gene (Supplementary Fig. 5f). Moreover, Hypergeometric Optimization of Motif EnRichment (HOMER) motif analysis based on ChIRP-seq data indicated the top enriched motif sequence of *MaTAR25* interacting genes is GGTGGTGGAGAT further supporting the *MaTAR25*-PURB-binding motif sequence (Supplementary Fig. 4a). Therefore, we performed chromatin immunoprecipitation (ChIP) using a PURB antibody and multiple qPCR primer pairs and showed that PURB has a high occupancy capacity over this region of *Tns1*. Importantly, the occupancy was impaired in *MaTAR25* KO cells and was able to be restored upon ectopic expression of *MaTAR25* in 4T1 *MaTAR25* KO cells (Fig. 5e). Moreover, we found the binding ability of *MaTAR25* to its downstream targeting regions (*Tns1, PVT1,* and *Has2*) was reduced in 4T1 PURB KO cells but not in PURA KO cells compared to 4T1 control cells using ChIRP-qPCR (Supplementary Fig. 5g). Together, these results provide compelling evidence indicating the interaction of PURB protein with *MaTAR25* is required for PURB binding to regulatory motifs in the *Tns1* gene in 4T1 cells.

### Human lncRNA *LINC01271* is the human ortholog of *MaTAR25*

**Fig. 6:**
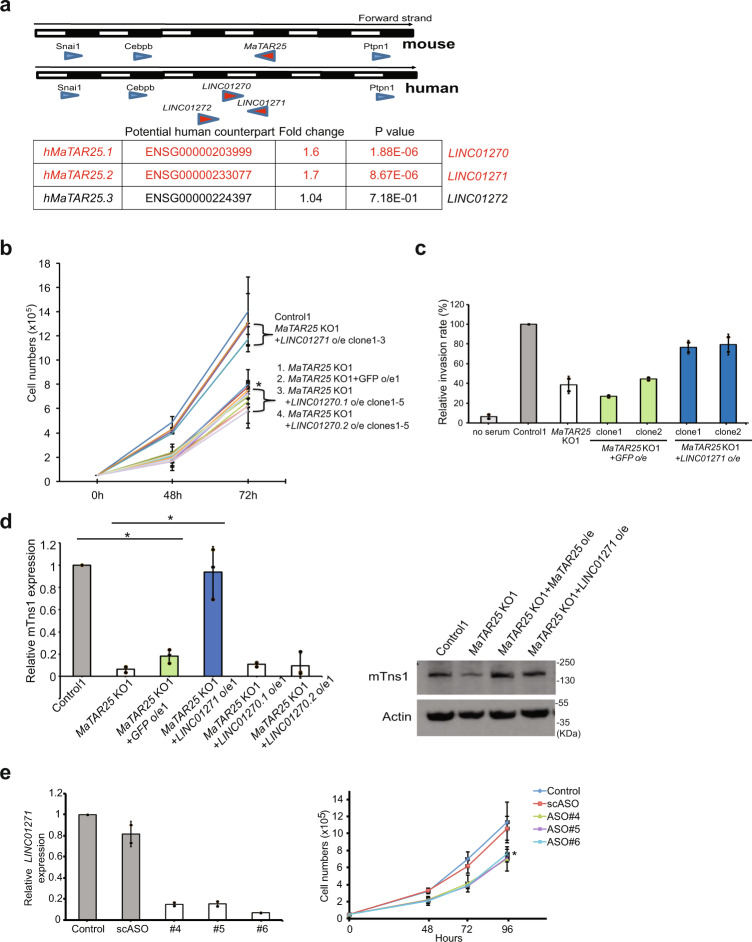
Human *LINC01271* is the human ortholog of *MaTAR25*. **a** All potential human orthologs of *MaTAR25* (*hMaTAR25*) were identified based on conservation of genomic location (synteny). RNA-seq data from The Cancer Genome Atlas (TCGA) was analyzed to evaluate the expression status of all potential *hMaTAR25* candidates by comparison of 1128 TCGA breast tumor datasets to 113 normal breast tissue controls. Fold change and statistical significance were calculated using the Wilcoxon rank test; *P* values were adjusted using the Benjamini & Hochberg method by DESeq2^30^. **b** Attempted rescue of 4T1 *MaTAR25* KO cells upon ectopic expression of two transcript isoforms of *LINC01270* (*LINC01270.1*, and *LINC01270.2*), or *LINC01271* in cell viability assays. The mean cell numbers of three independent replicates of 4T1 control1, *MaTAR25* KO1, *MaTAR25* KO1 with GFP, *LINC01270.1* clone1-5, *LINC01270.2* clone1-5, and *LINC01271* clone1-3 are shown ± SD (*n* = 3) *P < 0.05 (paired Student’s *t* test; two-tailed). **c** 4T1 *MaTAR25* KO cells with ectopic expression of GFP was used as a control to assess rescue in a cell invasion assay. The mean relative cell invasion of two independent replicates of 4T1 control, *MaTAR25* KO1, *MaTAR25* KO1 with GFP clone1-2, *LINC01271* ectopic expression clone1-2 is shown ± SD (*n* = 2 independent experiments). Ectopic expression of *LINC01271* can rescue the *MaTAR25* KO cell invasion phenotype. **d** RNA expression level of *Tns1* was determined in *MaTAR25* KO1 cells ectopically expressing *LINC01270.1*, *LINC01270.2*, or *LINC01271* by qRT-PCR. Data are presented as mean values ± SD (*n* = 3 independent experiments). *P < 0.05 (paired Student’s *t* test; two-tailed). The protein level of mTns1 was also examined in *MaTAR25* KO cells with ectopic expression of *LINC01271* by immunoblot analysis. **e** Three different ASOs targeting *LINC01271* were used to independently knockdown *LINC01271* in MDA-MB-231 LM2 cells. Left panel: the knockdown efficiency is calculated and data are shown as mean values ± SD (*n* = 2 independent experiments) by qRT-PCR after 24 h treatment of ASOs. Right panel: the mean cell numbers of three independent cell counting experiments of MDA-MB-231 LM2 mock-treated control cells, cells treated with scrambled ASO, and cells treated with three different *LINC01271* ASOs are shown ± SD (*n* = 3). *P < 0.05 (paired Student’s *t* test; two-tailed).

In order to translate our exciting findings in regard to mouse *MaTAR25* to the human system for potential future clinical applications, we went on to characterize the human ortholog of *MaTAR25* and confirm its function in human breast cancer cells. Based on syntenic conservation between the human and mouse genomes, we previously found three lincRNAs as potential human counterparts of *MaTAR25*: *LINC01270*, *LINC01271*, and *LINC01272*^30^ (Fig. 6a). Among these three lncRNAs, only *LINC01271* is transcribed in the same direction as *MaTAR25*. Analysis of The Cancer Genome Atlas (TCGA) data^30^ suggests two of these potential orthologs, *LINC01270* and *LINC01271*, are expressed at increased levels in multiple subtypes of breast cancer (Fig. 6a). Therefore, we focused on these two lincRNAs and performed independent ectopic expression of *LINC01270* and *LINC01271* in 4T1 *MaTAR25* KO cells to determine if one of these human lincRNAs could rescue the mouse *MaTAR25* KO phenotype. Cell viability assays indicated that ectopic expression of *LINC01271*, but not *LINC01270*, can rescue the proliferation phenotype of *MaTAR25* KO cells (Fig. 6b). Invasion assays also showed that ectopic expression of *LINC01271* in 4T1 *MaTAR25* KO cells can rescue the cell invasion phenotype (Fig. 6c). In addition, the expression of *Tns1* can also be restored to a similar level as in control 4T1 cells upon overexpression of *LINC01271* in *MaTAR25* KO cells (Fig. 6d). Immunoprecipitation (RIP) using the PURB antibody indicated a specific interaction between PURB and *LINC01271* (Supplementary Fig. 6a) in human triple-negative breast cancer MDA-MB-231 LM2 cells^45^. Next, we performed smRNA-FISH to examine the localization of *LINC01271* within MDA-MB-231 LM2 cells and we found that similar to *MaTAR25* it is a nuclear-enriched RNA (Supplementary Fig. 6b). Together, these results validate *LINC01271* as the human ortholog of *MaTAR25*.

### *LINC01271* may play a role in human breast cancer progression and have diagnostic and/or therapeutic potential

We performed ASO-mediated KD of *LINC01271* in MDA-MB-231 LM2 cells and selected the three most effective *LINC01271* ASOs to assess a KD phenotype. After 96 h of ASO treatment to mediate KD of *LINC01271* in MDA-MB-231 LM2 cells (Fig. 6e, left panel), all three independent ASOs decreased cell viability by ~32% (Fig. 6e, right panel). The KD result supports a role for *LINC01271* in human breast cancer progression.

By using reactome pathway analysis with *LINC01271* expression based on breast cancer TCGA data, we found the top pathways are related to the cell cycle (Supplementary Fig. 6c). In addition, according to the lncRNA database TANRIC^46^, higher expression of *LINC01271* is correlated with poor breast cancer patient survival (Supplementary Fig. 6d). qRT-PCR analysis of the expression level of *LINC01271* in breast tumor organoids vs organoids derived from normal adjacent breast tissue showed a higher expression level of *LINC01271* in tumor-derived organoids (Supplementary Fig. 6e).

**Fig. 7:**
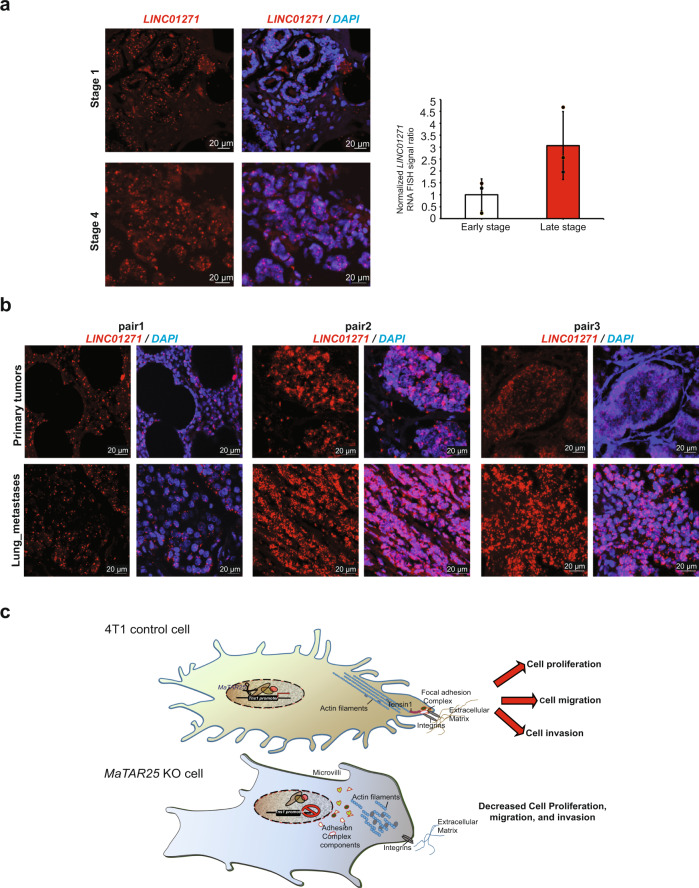
*LINC01271* expression in breast tumors and lung metastases. **a** smRNA-FISH images showing the expression of *LINC01271* (red) in patient breast tumor sections from different stages of breast cancer. More than six random areas on each slide were examined, and representative images are shown. Scale bars are 20 μm. The quantification of six images (three early stage and three late stage) of breast tumor sections is shown and data are presented as mean values ± SD. *P* = 0.08587 (paired Student’s *t* test; two-tailed). **b** smRNA-FISH images showing the expression pattern of *LINC01271* (red) within luminal subtype patient breast cancer primary tumors and lung metastases sections from the same patients. More than six random areas on each slide were examined, and representative images are shown. Scale bars are 20 μm. **c** Proposed model of *MaTAR25* function. *MaTAR25* acts as a scaffold and/or chaperone recruiting PURB to the *Tns1* gene where it induces its transcription. The Tns1 protein associates with focal adhesion complexes regulating signaling between the extracellular matrix and the actin cytoskeleton. KO or KD of *MaTAR25* results in a reorganization of the actin cytoskeleton and focal adhesion complexes as well as a significant decrease in microvilli resulting in a reduction in cell proliferation and migration.

Next, we performed smRNA-FISH to localize *LINC01271* in patient breast tumor sections. We found that *LINC01271* expression level was increased with increased breast tumor stage (Fig. 7a and Supplementary Fig. 7a), and we identified the presence of clonal and regional differential expression patterns in most of the breast tumor patient samples (Supplementary Fig. 7b). Most interestingly, lung and lymph node metastases exhibited higher expression of *LINC01271* than primary tumors from the same patients (Fig. 7b and Supplementary Fig. 7c). Thus, together these findings from patient-derived samples support our hypothesis that *LINC01271* is a potential therapeutic target to impact breast cancer progression and metastasis.

## Discussion

We identified *MaTAR25* as a lncRNA that is upregulated in HER2+, luminal, and TNBC as compared to normal mammary epithelial cells. Genetic KO or ASO KD of *MaTAR25* results in a reduction in cell proliferation, migration, and invasion (Fig. 7c). Injection of 4T1 *MaTAR25* KO cells into the mouse mammary fat pad or tail vein results in smaller tumor growth and a significant reduction in lung metastases. The human ortholog of *MaTAR25* was identified as *LINC01271*, and it is expressed in primary breast tumors and at even higher levels in lymph node and lung metastases. Increased expression of the human ortholog of *MaTAR25* is associated with poor patient prognosis^46^.

In order to understand the molecular mechanism by which *MaTAR25* imparts its function, we used ChIRP-seq and RNA-seq to identify and validate the *Tns1* gene as a direct downstream target of *MaTAR25*. *MaTAR25* positively regulates the expression of *Tns1* in mammary cancer cells through binding to its DNA sequence in *trans*. Tns1 has been shown to localize to focal adhesions and fibrilla adhesions, and to assist in mediating signaling between the extracellular matrix and the actin cytoskeleton to impact cell movement and proliferation^36,37^. Several reports have shown that loss of *Tns1* can cause a decrease in cell motility in many cell types^47–49^, and *Tns1* has also been shown to be involved in epithelial–mesenchymal transition (EMT) of cancer cells^50^. The general relationship between the expression of *Tns1* and different stages/subtypes of breast cancer has been unclear. However, Kaplan–Meier survival analysis indicates that the expression of *Tns1* is increased in grade 3 breast tumors^38^ supporting our findings of a positive role of *Tns1* in breast tumor progression mediated by *MaTAR25*. Although *Tns1* is the top target of *MaTAR25* with the highest statistical significance we cannot rule out the possibility that *MaTAR25* also regulates other genes as well given the additional candidates identified in our ChIRP-seq analysis. These candidates will be the focus of future studies.

By performing RNA antisense oligonucleotide pulldown (RAP) in 4T1 cells combined with iTRAQ mass spectrometry, to determine the relative quantitation of *MaTAR25*-associated proteins, we identified a specific interaction between *MaTAR25* and purine-rich element-binding protein B (PURB) which appears to be crucial for the downstream regulation of *MaTAR25*. PURB has been reported to be a transcriptional co-activator and binds to the single strand of the repeated purine-rich element PUR which is present in promoter regions and as such has been implicated in transcriptional control. PURB plays different roles in many physiological and pathological processes^51–53^. For example, PURB has been shown to be overexpressed in several different cancer types^54^. In addition, a previous study showed PURB to act as a transcriptional co-factor that can be recruited by *linc-HOXA1* to mediate the transcriptional regulation of *linc-HOXA1* in embryonic stem cells^44^ supporting a critical role of PURB with different lincRNAs in different biological contexts. We identified the *MaTAR25* target region of the *Tns1* gene to have a high ratio of purine to pyrimidine bases (3:1), and our ChIP-qPCR result indicated a high occupancy capacity of PURB over this targeting region, further supporting a functional role of PURB in partnering with *MaTAR25* to regulate the *Tns1* gene. The results of ectopic overexpression or siRNA-mediated knockdown of PURB, resulting in altered expression (upregulation or downregulation, respectively) of *Tns1* in 4T1 mammary cancer cells indicating a transcriptional regulatory role of PURB in this context. As the interaction of PURB with *MaTAR25* is essential for PURB binding to *Tns1* DNA this suggests that *MaTAR25* acts as a chaperone and/or scaffold for the *MaTAR25*/PURB/*Tns1* DNA complex, which is critical for transcriptional regulation of *Tns1* thereby impacting cancer progression.

PURB can form a homodimer, a heterodimer with PURA, or a heterotrimer with PURA and Y-box protein 1 (YBX1). Interestingly, these two additional proteins were also identified in our MS-iTRAQ analysis of *MaTAR25* interactors, and have been studied in many cancer types^55–57^. Future investigation of these potential interactions may provide further insights into the molecular mechanism of the *MaTAR25-* PURB complex in cancer cells.

Based upon synteny and further validation, we identified *LINC01271* as the human ortholog of *MaTAR25*. Interestingly, *LINC01271* has been identified as one of 65 new genetic loci that are related to overall breast cancer risk^58^. *LINC01271* expression is increased in breast cancer and correlates with poor clinical outcome based on the analysis of patient clinical data^46^. In addition, our results from examining sections of breast tumors showed a positive correlation between the expression level of *LINC01271* and the breast cancer stage. Our finding of an even higher level of expression in lung metastases from the same patients was especially interesting. Metastasis is the major cause of cancer-related deaths, particularly in breast cancer patients^59,60^, and developing an efficient treatment strategy to target and reduce breast cancer metastasis still remains the key challenge for this disease. Our ability to use ASOs to knockdown *MaTAR25* in the HER2/neu mouse model resulting in necrotic tumors and a significant reduction in metastasis has therapeutic implications. Using ASO targeting as a therapeutic approach has been applied to many diseases^61–63^ including cancer^26,64,65^ in recent years. A cEt ASO targeting the transcription factor STAT3 has shown robust single-agent activity in highly treatment-refractory lymphoma and non-small cell lung cancer studies^64^. The STAT3 ASO (AZD9150) has advanced into multiple Phase I and II clinical trials (NCT01563302, NCT02983578, NCT02549651). In addition, an antisense drug targeting all forms of the androgen receptor for the treatment of advanced metastatic prostate cancer has entered a clinical trial (NCT02144051). In this study, we developed three ASOs targeting *LINC01271* for functional assays in vitro. Ultimately, *LINC01271* is an ideal candidate to be exploited as a potential prognostic/therapeutic target and *LINC01271*-specific ASOs as therapeutics to impact breast cancer progression and metastasis.

## Methods

### Cell culture

Murine 4T1 cells, murine NF639 (cNeu; MMTV-Neu-NDL) cells, human MDA-MB-231 cells, and human MDA-MB-231 LM2 cells were cultured in DMEM with 10% FBS, and 1% penicillin/streptomycin. All cell lines were cultured in a cell culture incubator at 5% CO_2_ at 37 °C.

### Organoid culture

Surgically removed tumor samples from breast cancer patients along with adjacent normal tissue were collected from Northwell Health in accordance with Institutional Review Board protocol IRB-03-012 (TAP16-08). Informed consent ensured that the de-identified materials collected, the models created, and data generated from them can be shared without exceptions with researchers in the scientific community. Tumor and normal organoids were developed using a previously published protocol^66^. The tissues were manually cut into smaller pieces and treated with Collagenase IV (Sigma-Aldrich, C5138) at 37 °C. The samples were manually broken down by pipetting into smaller fragments and seeded in a dome of matrigel (Corning, 356231). Organoids were grown in culture media which contained 10% R-Spondin 1 conditioned media, 5 nM neuregulin 1 (Peprotech, 100-03), 5 ng/ml FGF7 (Peprotech, 100-19), 20 ng/ml FGF10 (Peprotech, 100-26), 5 ng/ml EGF (Peprotech, AF-100-15), 100 ng/ml noggin (Peprotech, 120-10 C), 500 nM A83-01 (Tocris, 2939), 5 μM Y-27632 (Abmole, Y-27632), 1.2 μM SB202190 (Sigma-Aldrich, S7067), 1× B27 supplement (Gibco, 17504-44), 1.25 mM N-acetylcysteine (Sigma-Aldrich, A9165), 5 mM nicotinamide (Sigma-Aldrich, N0636), 1× glutamax (Invitrogen, 12634-034), 10 mM HEPES (Invitrogen, 15630-056), 100 U/ml Pen-Strep (Invitrogen, 15140-122) 50 μg/ml Primocin (Invitrogen, ant-pm-1) in 1× advanced DMEM-F12 (Invitrogen, 12634-034)^66^. Cultures were passaged every 2–4 weeks using TrypLETM (Thermo Fisher, 12605028) to break down the organoids into smaller clusters of cells and re-plating them in matrigel.

#### Organoid RNA extraction

RNA was extracted using TRIzol^®^ (Thermo Fisher, 15596018) RNA extraction protocol followed by suspension in nuclease-free deionized water. Reagents used in this study are provided in Supplementary Data 6.

### Mice

All animal procedures and studies were approved by the Cold Spring Harbor Laboratory Animal Use Committee in accordance with IACUC procedures. Mice were maintained, and the experiments were performed with oversight of the Cold Spring Harbor Laboratory Animal Shared Resource, which is fully accredited by the Association for Assessment and Accreditation of Laboratory Animal Care (AAALAC). Briefly, male MMTV-Neu-NDL mice (FVB/N background) were kindly provided by Dr. William Muller (McGill University, Canada). Male MMTV-Neu-NDL mice were crossed with wild type FVB/N female mice purchased from The Jackson Laboratory for breeding. PCR genotyping was applied to select female mice with heterogeneous genotypes for MMTV-Neu-NDL for later in vivo ASO injection experiments. In all, 4–6-week-old BALB/c female mice were purchased from the Jackson Laboratory for in vivo 4T1 cell mammary fat pad (MFP) injection and tail-vein injection experiments.

### 5′/3′ rapid amplification of cDNA ends (RACE)

5′ and 3′ RACE of *MaTAR25* transcripts was performed on TRIzol-extracted RNA using the Invitrogen FirstChoice RLM-RACE kit (Thermo Fisher, AM1700M) according to the manufacturer’s instructions. Briefly, fragments were amplified by nested PCR using AmpliTaq Polymerase. PCR products were separated on 2% agarose, bands excised, gel purified, sub-cloned into pGEM-T Easy vector (Promega, A137A), and four or more clones per fragment were sequenced using standard Sanger sequencing. Primer sequences are provided in Supplementary Data 8.

### Northern blot analysis

The total RNA was isolated from MMTV-PyMT tumor cells using the TRIzol method, and the quality of the RNA sample (A260/A280 ratio) was measured using a Nanodrop 2000 (Thermo Fisher Scientific). *MaTAR25*-specific radiolabeled DNA probes were generated using dCTP ^32^P (PerkinElmer, BLU513H250UC) in a random primed labeling reaction. The total RNA was extracted by the TRIzol method. Analysis of RNA expression was performed by following the NorthernMax^®^ Kit manual (Thermo Fisher, AM8671, AM8676, and AM8677). Briefly, 20 μg and 30 μg of total RNA samples were denatured at 70° C for 10 min and electrophoresed in a 1% agarose gel (~4.5 h). The separated RNA was transferred from the gel to a positively charged nylon membrane (NC) by capillary blotting at room temperature overnight (or 16 h). The transferred RNA was then fixed to the NC membrane using UV cross-linking by Stratalinker on automode. The cross-linked membrane was prehybridized with ultrahyb-oligo hybridization buffer (Thermo Fisher, AM8663) and hybridized with the heat-denatured *MaTAR25*-specific radiolabeled DNA probe in a roller hybridization bottle at 42 °C overnight. After washing with SSC (G-Biosciences, R020) wash buffer by the steps: 2 × SSC with 1% SDS at 42 °C twice for 10 min each time, 1 × SSC with 0.5% SDS at 42 °C  twice for 10 min each time, and 1 × SSC with 0.1% SDS at 37 °C twice for 10 min each time, the wrapped membrane was exposed to a phosphorImager screen in a cassette or X-ray film (VWR, 95017-661) for signal detection. For stripping the membrane, the membrane was incubated in 50 ml 0.1% SDS solution (just to boiling; ~4 min in the microwave) in the roller hybridization bottle for 15 min. The SDS solution was discarded in a radioactive waste bottle. The steps were repeated again (total two washes), and the membrane was checked with a Geiger counter before re-probing. Reagents used in this study are provided in Supplementary Data 6.

### Cell lysate preparation for immunoblot analysis

Cells were trypsinized, and harvested cell pellets were lysed in RIPA buffer (Thermo Scientific, 89900; 25 mM Tris-HCl pH 7.6, 150 mM NaCl, 1% NP-40 substitute, 1% sodium deoxycholate, and 0.1% SDS) supplemented with 1× Roche protease inhibitor cocktail. The cell lysate was incubated on ice for 15 min, then sonicated for 5 min before centrifugation at 13,000 × *g*. The supernatant was collected and quantified using the Pierce™ BCA protein assay kit (Thermo Fisher, 23225).

### In vitro transcription/translation

T7 promoter containing DNA sequences or plasmids were used in a TNT^®^ Quick Coupled Transcription/Translation System (Promega, L1170) following the manufacture’s protocol. Briefly, 1 μg plasmid DNA template was mixed with 40 μl TNT^®^ T7 Quick Master Mix, 1 μl methionine (1 mM), 1 μl Transcend™ Biotin-Lysyl-tRNA, and nuclease-free water for the final volume of 50 μl per reaction. The reaction tube was incubated at 30 °C for 90 min, and 1 μl reaction product was added into diluted 2× Laemmli sample buffer (BIO-RAD, 1610737) for immunoblot analysis. Samples were loaded on 4–20% Mini-PROTEAN^®^ TGX™ Precast Protein Gels (BIO-RAD, 4561094), and the signals were detected by streptavidin–HRP (Jackson Immunoresearch, 016-030-084).

### RNA isolation and quantitative real-time PCR (qRT-PCR) assays

The total RNA was extracted using TRIzol following the manufacture’s protocol (* make sure to use nuclease-free reagents and tools). In general, 1 ml TRIzol was added for the RNA extraction to a 1.5-ml tube containing the cell pellet (5–10 × 10^6^) in the chemical hood. The sample was completely dissociated in the TRIzol reagent at room temperature for 5 min for homogenization. In all, 200 µl of chloroform per 1 ml of TRIzol reagent was added into the sample tube, and the sample tube was inverted ten times then incubated at room temperature for 3 min. The sample was centrifuged at 12,000 × *g* for 15 min at 4 °C for phase separation. The colorless upper aqueous phase containing RNA was transferred carefully to the new 1.5-ml tube, and 0.5 ml of isopropyl alcohol per 1 ml of TRIzol reagent and 1 µl GlycoBlue™ Coprecipitant (Thermo Fisher, AM9516) were added. The sample tube was inverted ten times and incubated at room temperature for 10 min or −20 °C overnight for RNA precipitation. The sample was centrifuged at 12,000 × *g* for 15 min at 4 °C. The blue color RNA pellet was visible on the bottom of the tube after centrifugation. After removing the supernatant, 1 ml of 75% ethanol per 1 ml of TRIzol reagent was added to the sample tube to wash the RNA pellet. The sample tube was vortexed for 10 s and centrifuged at 10,000 × *g* for 10 min at 4 °C. After centrifugation, the ethanol was removed completely and the RNA pellet was air-dried at room temperature but not dried completely (~10 min). The dried RNA pellet was dissolved in 10–20 µl Invitrogen nuclease-free water (Thermo Fisher, AM9932) and incubated at 50 °C in a shaking incubator for 10 min. The quality and quantity of the RNA sample were measured using a Nanodrop 2000 (Thermo Fisher Scientific) to check A260/A230 and A260/A280 ratios. RNA samples with 1.8–2.0 A260/A280 ratios were used as high-quality samples. In all, 1 μg of the total RNA was treated with DNaseI (Thermo Fisher, 18068015) and reverse transcribed into cDNA using TaqMan Reverse Transcription Reagent kit (Thermo Fisher, 4304134), followed by qPCR with SYBR green PCR master mix (Thermo Fisher, A25743) on an ABI QuantStudio 6 Flex Real-Time PCR System. qRT-PCR conditions were as follows: 30 min at 50 °C for reverse transcription, 15 min at 95 °C for the initial activation step followed by 40 cycles of 15 s at 94 °C, 30 s at 60 °C. Mouse peptidylprolyl isomerase B (cyclophilin B; Ppib) and human GAPDH and RPL13A were used as endogenous controls to normalize each sample. Reagents used in this study are provided in Supplementary Data 6, and primer sequences are provided in Supplementary Data 8.

### Cell fractionation, cytoplasmic/nucleoplasmic/chromatin-related RNA isolation

Cell fractionation was done using a standardized protocol^67^. After reaching 80% confluence, cultured 4T1 cells were trypsinized and counted. In total, 3 × 10^6^ 4T1 cells were collected and lysed in NP-40 substitute (Thermo Fisher, AAJ19628AP) lysis buffer (10 mM Tris pH 7.5, 150 mM NaCl, and 0.15% NP-40 substitute) supplemented with SUPERase-In RNase Inhibitor (Thermo Fisher, AM2696) on ice for 5 min. Ten percent of the cell lysate was collected into a new 1.5-ml tube on ice for RNA extraction later as the reference input. Ninety percent of the cell lysate was overlaid on top of sucrose buffer (10 mM Tris pH 7.4, 150 mM NaCl, and 24% sucrose) and centrifuged at 3500 × *g* for 10 min to separate the cytoplasmic fraction and nuclei. The supernatant was transferred to a new 1.5-ml tube as the cytoplasmic fraction, and the pellet as the nuclei. Next, the nuclear pellet was rinsed with PBS-EDTA once and resuspended with glycerol buffer (20 mM Tris pH 7.4, 75 mM NaCl, 0.5 mM EDTA, and 50% glycerol) supplemented with SUPERase-In RNase Inhibitor mixed with urea buffer (1 M Urea, 0.3 M NaCl, 7.5 mM MgCl_2_, 0.2 mM EDTA, and 1% NP-40 substitute) on ice for 2 min. The lysate was then centrifuged at 13,000 × *g* for 2 min to separate the nucleoplasmic fraction and chromatin pellet. The chromatin was resuspended in TRIzol reagent and fully solubilized by passing through the 21-gauge needle/syringe. The cytoplasmic fraction and nucleoplasmic fractions were also used for RNA extraction using TRIzol reagent. RNA extracted from different fractions was applied for cDNA synthesis and qRT-PCR. Primer sequences are provided in Supplementary Data 8.

### CRISPR/Cas9 genetic knockout

To generate a genetic knockout of *MaTAR25*, two sgRNAs targeting the promoter region were combined, creating a deletion including the TSS. Both sgRNAs were designed using http://crispr.mit.edu/. The sgRNA targeting the gene body of *MaTAR25* was cloned into a pSpCas9(BB)-2A-GFP vector (Addgene, 48138), and the sgRNA targeting the upstream promoter region was cloned into a pSpCas9(BB)- 2A-mCherry vector. 4T1 cells were transfected with both plasmids using SE Cell Line 4D-Nucleofector kit (Lonza, V4XC-1024) in 4D-Nucleofector X Unit (Lonza) with program code “CN-114”. Cells transfected with a sgRNA targeting *Renilla* luciferase were used as a negative control. To select for cells expressing both gRNAs, GFP and mCherry double-positive cells were sorted 40-h post transfection, as single-cell deposition into 96-well plates using a FACS Aria (SORP) Cell Sorter (BD) (Supplementary Data 10). Each single-cell clone (control and *MaTAR25* KO) was propagated and analyzed by genomic PCR and qRT-PCR to select for homozygous knockout clones.

To generate a genetic knockout of Tns1, PURB, and PURA, sgRNAs targeting different exons of genes were designed using http://crispr.mit.edu/. The sgRNAs were cloned into a pSpCas9(BB)-2A-Puro (PX459) V2.0 vector (Addgene, 62988). 4T1 cells were transfected using SE Cell Line 4D-Nucleofector kit (Lonza, V4XC-1024) in 4D-Nucleofector X Unit (Lonza) with program code “CN-114”. Cells transfected with a sgRNA targeting *Renilla* luciferase were used as a negative control. Transfected cells were placed under 2 μg/ml puromycin (Sigma-Aldrich, P8833) in culture media for selection. Selected pool cells were propagated and analyzed by qRT-PCR and western blotting to confirm. Assays and resources used in this study are provided in Supplementary Data 7, and sequences for all sgRNAs and primers are provided in Supplementary Data 8.

### Cell counting viability assay

After reaching 80% confluence in a 100-mm culture dish, cells were trypsinized and harvested. Cells were resuspended and applied to a hemocytometer for manual counting. The same number of cells (2.5 or 5 × 10^4^) of each group was seeded into each well of a six-well tissue culture plate at day 0 and then seeded cells were incubated in a 37 °C incubator with 5% CO_2_. At different time points of incubation, cells were trypsinized and harvested for manual counting. Trypan Blue-treated cell suspensions were collected and applied to a hemocytometer for manual counting.

### Cell cycle analysis

Cell cycle analysis was performed using BD bromodeoxyuridine (BrdU) FITC assay kit (BD, 559619) following the manufacturer’s protocol. Briefly, cultured cells were incubated with BrdU containing medium for 30 min, and FITC-conjugated anti-BrdU antibody was applied for labeling actively synthesizing DNA. 7-aminoactinomycin D (7-AAD) was used for labeling total DNA. The labeled cell samples were analyzed on the BD LSR II flow cytometer, and BrdU FITC-A vs DNA 7-AAD dot plot with gates was used to encompass the G0/G1, S, and G2/M populations. The collected cytometry data were analyzed with FACSDiva™ and FlowJo software.

### Migration assay

Live-cell tracking was performed to examine cell migration over time. After reaching 80% confluence on 100-mm culture dish, cells (4T1 control clones and 4T1 *MaTAR25* KO clones) were harvested and the same number of cells (2 × 10^4^) were seeded into six-well tissue culture plates at day 0. Cells were cultured in a 37 °C cell culture incubator at 5% CO_2_ for 12 h before imaging.

Cells were imaged in the microscope chamber at 37 °C with 5% CO_2_, and images were collected every 5 min (five viewpoints were selected from each well) using a Zeiss AxioObserver microscope for 8 h. The images of the individual samples were converted to videos using ImageJ. Videos were analyzed by CellTracker image-processing software (https://academic.oup.com/bioinformatics/article/32/6/955/1744578), and the migration distance (μm or pixels) of cells were measured by manual and semi-automatic tracking. The mean relative migration distance (μm or pixels) of three independent replicates of 4T1 control clone groups and *MaTAR25* KO clone groups were calculated.

### Scratch wound-healing assay

Cultured cells were harvested and seeded into each well of a 24-well tissue culture plate. Cells were incubated until they reached at least 90% confluence. The wound line was created by “scratch” with a p200 micropipette tip, and cells were washed with PBS twice to remove the debris and smooth the edge of the scratch. To create markings close to the wound line as reference points for imaging, we labeled the outer bottom of the dish with a marker pen. At least two areas of each well were imaged immediately as the time point 0 h, and culture plates were placed back in the incubator at 37 °C for 8–24 h. During the incubation, the plates were brought out for imaging every 6–8 h and returned to the incubator to determine the actual endpoint for specific cell types. For 4T1 cells, the endpoint of the scratch wound-healing assay is 12 h. After 12 h of incubation, the same areas based on the reference points of each well were imaged again, and the migration areas in each well were measured by ImageJ for comparison.

### Invasion assay

Invasion assays with 4T1 *MaTAR25* knockout cells and 4T1 control cells were carried out using the Cultrex^®^ 24-well BME Cell Invasion Assay (Trevigen, 3455-024-K) following the manufacturer’s protocol. BME coating: The day before the experiment, the inserts and culture well plates were assembled as assay chambers, and 100 µl of 0.1–1× BME stock solution was prepared as the coating solution (for 4T1 cells, use 0.5× BME stock solution) for each insert. Coated chambers were incubated at 37 °C overnight. Starvation: After reaching 70% confluence in 100-mm culture dish, cells were washed with PBS twice, and serum-free DMEM medium once. Cells were starved by incubating in serum-free DMEM medium for 12 h before the experiment. Incubation for cell invasion: Cells were harvested and resuspended using serum-free DMEM medium. After counting, cells were diluted and seeded at a density of 1 × 10^5^ cells in 100 µl serum-free DMEM medium per well into the invasion assay chamber. Just before seeding cells, the coating solution was carefully aspirated from the insert without touching the membrane. In all, 500 µl of culture medium (DMEM with 10% FBS) was added to the bottom chambers, and serum-free DMEM medium was used that did not stimulate cell invasion through the BME as a negative control. The plate was incubated at 37 °C for 24 h, and the assay was performed (incubation times for the assay varied from 4 h to 48 h for different cell types, and needed to be determined before the actual experiment). Measurement of invaded cells: After 24 h of incubation, the upper medium was removed, and cells from the top of inserts were washed with 100 µl 1× wash buffer without puncturing the membrane. The upper part of inserts was cleaned again using cotton tipped swabs gently without breaking the membrane. The insert was taken out, and the medium was aspirated from bottom chamber, and wash each well with 500 µl 1× wash buffer. The wash buffer was aspirated; 500 μl of Cell Dissociation Solution/Calcein-AM was added to the bottom chamber of each well. The inserts were added back to the plates, and the plates were incubated at 37 °C in a 5% CO_2_ incubator for 1 h (gently swirl the plates once during the incubation). After 1 h of incubation, the invaded 4T1 cells through the BME layer and attached to the bottom of the invasion insert were collected using cell dissociation solution and stained with Calcein-AM as the assay solution. The upper insert was removed, and the fluorescence of the assay solution was measured with a SpectraMax i3 Multi-Mode Detection Platform (Molecular Devices) using the Ex480/Em520 nm filter set.

### Cloning

Specific gene overexpression plasmids were constructed using NEBuilder HiFi DNA Assembly (NEB, E5520S) following the manufacture’s protocol. PCR products were cloned into pCMV6-entry plasmids digested with Sgf I and Fse I. Assembled plasmids were introduced into NEB stable competent *E.* *coli* using heat shock transformation and kanamycin selection. Four or more colonies per plate were picked and sequenced using standard Sanger sequencing.

### Antisense oligonucleotide (ASO) and siRNA-mediated knockdown (KD)

Specific 16mer antisense oligonucleotides (ASOs) comprised of phosphorothioate-modified short S-cEt (S-2′-O-Et-2′,4′-bridged nucleic acid) gapmer chemistry targeting *MaTAR25* and *LINC01271* were designed and provided by Ionis Pharmaceuticals, Inc. Briefly, cultured cells were harvested and seeded into culture dishes. Transfection-free uptake of ASOs was accomplished by adding 4 μM of either *MaTAR25/LINC01271*-specific ASOs or scrambled ASO (scASO) to the culture medium immediately after seeding the cells. Cells were incubated for indicated time points, and RNA was isolated using TRIzol reagent for qRT-PCR to check the knockdown efficiency. For siRNA-mediated knockdown, siRNAs (27-mers) targeting mouse PURB were purchased from ORIGENE (ORIGENE, SR410793), and siRNA transfection was done using Lipofectamine 2000 (Thermo Fisher, 11668030) following the manufacture’s protocol. RNA was extracted at different time points for qRT-PCR to check the knockdown efficiency. ASO sequences and primer sequences are provided in Supplementary Data 8.

### Chromatin isolation by RNA purification (ChIRP)-Seq

For Chromatin Isolation by RNA Purification (ChIRP), we followed a previously described protocol^35^. Briefly, 20 million cells were harvested and fixed in 1% glutaraldehyde solution (Sigma-Aldrich, G5882) for each reaction. ChIRP was performed using biotinylated oligo probes designed against mouse *MaTAR25* using the ChIRP probe designer (Biosearch Technologies). Independent even and odd probe pools were used to ensure lncRNA-specific retrieval (refer to Supplementary Data 8 for odd and even sequences targeting *MaTAR25*, and probes targeting mouse Ppib transcripts which were used as negative controls). ChIRP-Seq libraries were constructed using the Illumina TruSeq ChIP Library Preparation Kit (Illumina, IP-202-1012). Sequencing libraries were barcoded using TruSeq adapters and sequenced on Illumina NextSeq instruments.

### ChIRP-Seq data analysis

Data quality was assessed using FastQC (http://www.bioinformatics.babraham.ac.uk/projects/fastqc/) and paired-end reads were mapped to GRCm38 using Bowtie2^68^ with parameters --end-to-end --sensitive --fr, resulting in a 90% or higher overall alignment rate. ChIRP-seq analysis was performed using HOMER^69^. Differential ChIRP peaks were called using the getDifferentialPeaksReplicates.pl script, with negative control (Ppib) pull-down samples as background and parameters -style histone -f 50. Peaks identified with at least a 50-fold enrichment were processed further using the annotatePeaks.pl script and the GENCODE vM16 annotation. Both known and de novo motif analysis were carried out with the findMotifsGenome.pl script on the repeat-masked GRCm38 genome, +/− 500 bp around the identified ChIRP peaks.

### RNA-seq library construction

RNA was extracted using TRIzol following the manufacture’s protocol. RNA quality was assayed by running an RNA 6000 Nano chip (Agilent, 5067-1511) on the Agilent 2100 Bioanalyzer. In total,1 μg of the total RNA was used for constructing each RNA-seq library using the Illumina TruSeq sample prep kit v2 (Illumina, RS-122-2001) following the manufacturer’s protocol. Briefly, RNA was polyA selected and enzymatically fragmented. cDNA was synthesized using Super Script II master mix (Thermo Fisher, 18064014), followed by end repair, A-tailing, and PCR amplification. Each library was high-throughput single-end sequenced on Illumina NextSeq instruments.

### RNA-Seq data analysis

Data were analyzed as previously described^30^. Briefly, the quality of FASTQ files was assessed using FastQC (http://www.bioinformatics.babraham.ac.uk/projects/fastqc/). Reads were mapped to GRCm38 using STAR^70^, and the reads per gene record were counted using HTSeq-count^71^ and the GENCODE vM5 annotation. Differential gene expression was performed with DESeq2^72^, and a FDR-adjusted *P* value of <0.1 was set as a threshold for statistical significance. KEGG pathway and GO term enrichment and was carried out using the R/Bioconductor packages GAGE^73^ and Pathview^74^.

### RNA antisense pulldown for mass spectrometry

Cells were lysed in a 10-cm culture dish in 1 ml of IPLB, Pierce™ IP Lysis Buffer (Thermo Fisher, 87787; 25 mM Tris-HCl pH 7.4, 150 mM NaCl, 1% NP-40, 1 mM EDTA, 5% glycerol) supplemented with 100 U/ml SUPERase-IN (Thermo Fisher, AM2696) and 1× Roche protease inhibitor cocktail (Sigma-Aldrich, 11836170001) for 10 min, and the lysate was centrifuged at 13,000 × *g* for 10 min. The cell lysate was adjusted to 0.3 mg/ml (Pierce™ BCA Protein Assay). A total of 100 pmol of biotinylated oligo was added to 500 μl of lysate and incubated at room temperature for 1 h with rotation. In all, 100 μl of streptavidin Dynabeads were washed in IPLB, added to the lysate, and incubated for 30 min at room temperature with rotation. Beads were washed three times with 1 ml lysis buffer. For determining temperature for optimal elution, beads were then resuspended in 240 μl of 100 mM TEAB and aliquoted into eight PCR tubes. The temperature was set on a veriflex PCR block and incubated for 10 min. Beads were captured, and TRIzol was added to the eluate and beads. Once the optimal temperature is established, the beads were resuspended in 90 μl of 100 mM TEAB, and incubated at 50° C for 10 min. TRIzol was added to 30 μl of the eluate, another 30 μl was kept for immunoblots, and the last 30 μl aliquot was sent directly to the Cold Spring Harbor Laboratory Mass Spectrometry Shared Resource.

### Mass spectrometry

Tryptic digestion and iTRAQ labeling—Eluted samples were reduced and alkylated with 5 mM DTT and 10 mM iodoacetamide for 30 min at 55 °C, then digested overnight at 37 °C with 1 μg Lys-C (Promega, VA1170) and dried in vacuo. Peptides were then reconstituted in 50 μl of 0.5 M TEAB/70% ethanol and labeled with 4-plex iTRAQ reagent for 1 h at room temperature essentially as previously described^75^. Labeled samples were then acidified to <pH 4 using formic acid, combined and concentrated in vacuo until ~10 μl remained.

Two-dimensional fractionation—Peptides were fractionated using a Pierce™ High pH Reversed-Phase Peptide Fractionation Kit (Thermo Scientific, 84868) according to the manufacturer’s instructions with slight modifications. Briefly, peptides were reconstituted in 150 μl of 0.1% TFA, loaded onto the spin column, and centrifuged at 3000 × *g* for 2 min. Column was washed with water, and then peptides were eluted with the following percentages of acetonitrile (ACN) in 0.1% triethylamine (TEA): 5%, 7.5%, 10%, 12.5%, 15%, 20%, 30%, and 50%. Each of the 8 fractions was then separately injected into the mass spectrometer using capillary reverse-phase LC at low pH.

Mass spectrometry—An Orbitrap Fusion Lumos mass spectrometer (Thermo Scientific), equipped with a nano-ion spray source was coupled to an EASY-nLC 1200 system (Thermo Scientific). The LC system was configured with a self-pack PicoFrit™ 75-μm analytical column with an 8-μm emitter (New Objective, Woburn, MA) packed to 25 cm with ReproSil-Pur C18-AQ, 1.9 μM material (Dr. Maish GmbH). Mobile phase A consisted of 2% acetonitrile; 0.1% formic acid and mobile phase B consisted of 90% acetonitrile; 0.1% formic acid. Peptides were then separated using the following steps: at a flow rate of 200 nl/min: 2% B to 6% B over 1 min, 6% B to 30% B over 84 min, 30% B to 60% B over 9 min, 60% B to 90% B over 1 min, held at 90% B for 5 min, 90% B to 50% B over 1 min and then flow rate was increased to 500 μl/min as 50% B was held for 9 min. Eluted peptides were directly electrosprayed into the MS with the application of a distal 2.3 kV spray voltage and a capillary temperature of 300 °C. Full-scan mass spectra (Res = 60,000; 400–1600 m/z) were followed by MS/MS using the “Top Speed” method for selection. High-energy collisional dissociation (HCD) was used with the normalized collision energy set to 35 for fragmentation, the isolation width set to 1.2 and a duration of 15 s was set for the dynamic exclusion with an exclusion mass width of 10 ppm. We used monoisotopic precursor selection for charge states 2+ and greater, and all data were acquired in profile mode.

### Database searching

Peaklist files were generated by Proteome Discoverer version 2.2.0.388 (Thermo Scientific). Protein identification was carried out using both Sequest HT^76^ and Mascot 2.5^77^ against the UniProt mouse reference proteome (57,220 sequences; 26,386,881 residues). Carbamidomethylation of cysteine, iTRAQ4plex (K), and iTRAQ4plex (N-term) were set as fixed modifications, methionine oxidation, and deamidation (NQ) were set as variable modifications. Lys-C was used as a cleavage enzyme with one missed cleavage allowed. Mass tolerance was set at 20 ppm for intact peptide mass and 0.3 Da for fragment ions. Search results were rescored to give a final 1% FDR using a randomized version of the same Uniprot mouse database, with two peptide sequence matches (PSMs) required. iTRAQ ratio calculations were performed using Unique and Razor peptide categories in Proteome Discoverer.

### RNA immunoprecipitation (RIP)

RIP was performed following the Abcam protocol with minor modifications. 4T1 cells in culture were trypsinized and harvested, total 40 million cells were washed once with cold PBS before RIP experiment, then the cells were resuspended in 8 ml PBS, 8 ml nuclear isolation buffer (1.28 M sucrose, 40 mM Tris-HCl pH 7.5, 20 mM MgCl_2_, and 4% Triton X-100 supplemented with 100 U/ml SUPERase-IN and 1× Roche protease inhibitor cocktail), and 24 ml nuclease-free water on ice for 20 min with frequent mixing. The cleared lysates were pelleted by centrifugation at 2500 × *g* for 15 min. Pellets resuspended in 4 ml RIP buffer (150 mM KCl, 25 mM Tris pH 7.4, 5 mM EDTA, 0.5 mM DTT, and 0.5% NP-40 substitute supplemented with 100 U/ml SUPERase-IN and 1X Roche protease inhibitor cocktail) and sonicated for 5 min using BioRuptor Pico water bath sonicator (30 s ON/OFF) at 4 °C. The lysates were cleaned by centrifugation at 13,000 × *g* for 10 min. The supernatant was collected and separated, then incubated with 4 μg PURB antibody (Proteintech, 18128-1-AP), PURA antibody (Abcam, ab125200), or rabbit isotype IgG control for 2 h to overnight at 4 °C with gentle rotation. In all, 80 μl of protein A beads (Thermo Fisher, 88845) for rabbit antibody were added into the reactions and incubate for 1 h at 4 °C with gentle rotation. After washing three times with RIP buffer and once with PBS, beads were collected for immunoblot analysis and RNA extraction for qRT-PCR. Primers for RIP qRT-PCR can be found in Supplementary Data 8.

### Western blot analysis

SDS Polyacrylamide gel electrophoresis (SDS-PAGE): Boiled samples were loaded to 10% a home casting polyacrylamide gel or Mini-PROTEAN TGX Precast Protein Gels (BIO-RAD) for protein separation. Transfer: Proteins were transferred from the gel to 0.2-µm PVDF membrane (the PVDF membrane was pre-activated using 100% methanol) using a wet transfer method. For most target proteins (MW between 14 and 116 kDa), the transfer setting was 100 V for 70 min using 1× Tris-glycine transfer buffer supplemented with 20% methanol; for tensin1, the transfer setting was 30 V overnight using 1× Tris-Glycine transfer buffer supplemented with 10% methanol and 0.01% SDS. Blocking: Before the process, the membrane was stained with Ponceau S for 1 min to check the transferred pattern, and de-stained completely with 1× PBS with 0.1% Tween 20 (PBST). Then the membrane/blot was placed in blocking buffer (PBST with 5% nonfat milk) on an orbital shaker at room temperature for 1 h for blocking. Primary antibody incubation: Before the incubation, the blot was rinsed with PBST once. The blot was incubated in the primary antibody diluted in PBST on the orbital shaker at 4 °C overnight. The primary antibody dilution used in this study: 1:1000–1500 for Tensin1 antibody, 1:200 for PURA, 1:200 for PURB, and 1:5000–10,000 for actin antibody. Second antibody incubation: The blot was extensively washed with PBST three times for 10 min each on the orbital shaker with gentle agitation. After washing, the blot was placed in the secondary antibody diluted in PBST at room temperature for 1 h The HRP-conjugated secondary antibody dilution: 1:3000 for goat-anti-rabbit secondary antibody targeting tensin1 antibody, and 1:10,000–20,000 for goat-anti-mouse secondary antibody targeting actin antibody. ECL detection: The blot was extensively washed with PBST three times for 10 min each on the orbital shaker with gentle agitation. After washing, the blot was incubated with Pierce™ ECL Western Blotting Substrate (Thermo Scientific, PI32209) at room temperature for 1 min with gentle agitation (* light sensitive). The signal on the blot was detected by using X-ray film. Antibodies used in this study are provided in Supplementary Data 5.

### Chromatin immunoprecipitation (ChIP) coupled with quantitative PCR (ChIP-qPCR)

For Chromatin immunoprecipitation (ChIP), we followed a published protocol^78^. Cross-linking: 30 million 4T1 cells were harvested and cross-linked in 1% formaldehyde at room temperature for 20 min, then the reaction was quenched using 0.125 M glycine (Thermo Fisher, AAJ16405A7) at room temperature for 10 min. Samples were centrifuged at 2000 × *g* for 5 min at 4 °C, and cell pellets were collected. Sonication and immunoprecipitation (IP): Cell pellets were incubated with cell lysis buffer (10 mM Tris pH 8.0, 10 mM NaCl, 0.2% NP-40 substitute) for 15 min on ice. The cell lysates were quickly centrifuged at 2500 × *g* for 30 s at 4 °C, and the nuclei pellets resuspended in 1.5 ml of nuclei lysis buffer (50 mM Tris pH 8.0, 10 mM EDTA, 1% SDS) and incubated for 10 min on ice. Next, the samples were sonicated for 15 min using a BioRuptor Pico water bath sonicator (30 s ON/OFF) at 4 °C. The sonicated samples were centrifuged at max speed for 15 min at 4 °C, and supernatants were transferred into a new 1.5-ml tube as sonicated chromatin samples. Before IP, 5% of the sample was transferred into a 1.5-ml tube as Protein INPUT, and 5% of the sample was transferred into a 1.5-ml tube as DNA INPUT. For one IP, 1.5 ml of sonicated chromatin from 30 million cells were diluted with 21 ml IP-Dilution buffer (20 mM Tris pH 8.0, 2 mM EDTA, 150 mM NaCl, 1% Triton X-100, 0.01% SDS,) and incubated with 4 μg of PURB antibody or rabbit isotype IgG control. The sample tubes were incubated for 3 h 4 °C with rotation, and 80 μl of protein A beads for rabbit antibody was added into the sample tubes. Next, the samples were incubated at 4 °C overnight with rotation. Washing: After incubation, beads were separated and collected using a DynaMag™-2 Magnet stand. Beads were washed once with IP-wash 1 buffer (20 mM Tris pH 8.0, 2 mM EDTA, 50 mM NaCl, 1% Triton X-100, 0.1% SDS), twice with high-salt buffer (20 mM Tris pH 8.0, 2 mM EDTA, 500 mM NaCl, 1% Triton X-100, 0.01% SDS), once with IP-wash 2 buffer (10 mM Tris pH 8.0, 1 mM EDTA 0.25 M LiCl, 1% NP-40 substitute, 1% sodium deoxycholate), twice with TE buffer (10 mM Tris-Cl, 1 mM EDTA, pH 8.0). Elution and reverse cross-linking: Bead-bound chromatin was eluted in 800 μl nuclear lysis buffer by heating at 65 °C for 15 min. 48 μl of 5 M NaCl was added to the 800 μl of eluted chromatin, followed by incubation at 65 °C overnight for reverse cross-linking. DNA purification and analysis: After reverse cross-linking, DNA was treated with RNaseA (Sigma-Aldrich, R4642) and proteinase K (Viagen Biotech, 501-pk), followed by purification using QIAGEN PCR purification kit (QIAGEN, 28104). qPCR was performed on a ABI QuantStudio 6 Flex Real-Time PCR System. ChIP-qPCR primers can be found in Supplementary Data 8.

### Single-molecule RNA fluorescence in situ hybridization (FISH)

For single-molecule RNA-FISH, custom Type-6 primary probes targeting *MaTAR25*, *LINC01271,* and other lncRNAs were designed and synthesized by Affymetrix. For RNA-FISH on cultured cell samples, Affymetrix View ISH Cell Assay Kit (Thermo Fisher, QVT0050) reagents were used. Cultured cells were harvested and seeded onto acid-cleaned #1.5 glass coverslips (Electron Microscopy Sciences, 72230-01) for 24-h incubation to 70% confluence, cell samples then were fixed in freshly prepared 4% paraformaldehyde (PFA) (Electron Microscopy Sciences, 19200). Cells were then permeabilized and protease digested before hybridization. For RNA-FISH on formalin-fixed paraffin-embedded (FFPE) tissue sections of breast tumors and metastases, Affymetrix ViewRNA ISH Tissue 1-Plex Assay kit (Thermo Fisher, QVC0001) reagents were applied. Sections on slides were deparaffinized, protease digested and fixed with 10% NBF before hybridization. QuantiGene ViewRNA probe hybridizations were performed at 40 °C for 3 h. The hybridization and signal amplification steps were performed according to the manufacturer’s instruction, and nuclei were counterstained with DAPI (Thermo Fisher, D1306). Coverslips and tissue sections were mounted in ProLong Gold Antifade mounting medium (Thermo Fisher, P36930) before detection. Imaging was performed on Zeiss LSM 710/780 Confocal Microscope systems. Assays and resources used in this study are provided in Supplementary Data 7.

### DNA FISH

Different mouse BAC clones (RPCI-23) were used as template included (*MaTAR25*), and (*Tensin1*). In all, 1 μg of BAC DNA was used as a template for random priming reaction to generate amine-modified DNA, and amine-modified DNA was labeled with a reactive fluorescent dye as fluorescent probes according to the protocol provided with ARES™ Alexa Fluor™ DNA Labeling Kit (Thermo Fisher, A21665 and A21669). For DNA FISH, we followed protocols^79^. Briefly, cultured cells were seeded onto 22-mm^2^ glass coverslips (Corning) or etched grid coverslips (Bellco, 1916-92525), and incubated to 70% confluence in the 5% CO_2_ incubator at 37 °C. Coverslips were fixed with freshly prepared 4% PFA for 20 min at room temperature, and permeablized in 0.5% Triton X-100/1X PBS for 5 min on ice. Probes were prepared for hybridization by mixing 2 μl of probe with 5 μl each of sheared salmon sperm DNA (Thermo Fisher, AM9680), mouse Cot1 DNA (Thermo Fisher, 18440016), and yeast tRNA (Sigma-Aldrich, R5636), dehydrating the probe mixture in the speed-vac, and then resuspending the probe in 10 μl of deionized formamide (Thermo Fisher, AM9342). Just prior to hybridization, the probes were denatured at 95 °C for 10 v, transferred to ice for 5 v, and then mixed with 10 μl of 2× hybridization buffer (4× SSC, 20% dextran sulfate) and pipetted onto slides so that coverslips could be placed cell-side-down on the probe mixture for the hybridization reaction. Coverslips were sealed with rubber cement, and hybridization slides were placed in a humid chamber and incubating at 37 °C overnight. Post-hybridization washing: Before washing, prepare 37 °C 50% deionized formamide/2× SSC (pH 7.0) washing solution 1 in a glass jar, and prepare 37 °C 10 ml 2× SSC and 37 °C 10 ml 1× SSC as washing solution 2 and 3. Coverslips were removed from the hybridization slides and transferred to the glass jar containing washing buffer 1, and coverslips were incubated in a 37 °C water bath for 30 min with gentle shaking. Coverslips were removed from washing solution 1 and put into 2× SSC for washing at 37 °C for 15 v. Next, coverslips were placed into 1× SSC for washing at 37 °C for 15 min, equilibrated in 4× SSC at room temperature for 5 min. After these washing steps, nuclei were counterstained with DAPI. Coverslips were mounted in ProLong Gold Antifade mounting medium before detection. Coverslips were imaged using Zeiss LSM 710/780 Confocal Microscope systems.

### Immunofluorescence (IF)

For IF, we followed the protocol^80^ with minor modifications depend on applied antibodies. Cultured cells were harvested and seeded onto acid-cleaned #1.5 glass coverslips for 24-h incubation to 70% confluence, cell samples then were fixed in 4% formaldehyde for 20 min. Samples were permeabilized in 0.2% Triton X-100 plus 1% bovine serum albumin (BSA) (Sigma-Aldrich, A2153) in PBS for 5 min on ice. After incubated in 1% BSA in PBS for 30-min blocking, samples were incubated in the appropriate concentration (1:100 for paxillin, 1:100 for vinculin) of primary antibody for 1–2 h at room temperature, and incubated in diluted (1:200–1:400) secondary antibody solution (Alexa 594 conjugated) (Invitrogen, A-11012) with Phalloidin-iFluor 488 Reagent (stock solution:1000×; Abcam, ab176753) for 1 h in a humidified chamber at room temperature. After several washes, nuclei were counterstained with DAPI. Coverslips were mounted in ProLong Gold Antifade mounting medium before detection. Coverslips were imaged on the Zeiss LSM 710/780 Confocal Microscope systems.

### Transmission electron microscopy (TEM)

Cultured cells were harvested and seeded onto 10-cm culture dishes for 24 h, and fixed with 2.5% glutaraldehyde (EM grade) in 0.1 M phosphate buffer pH 7.4 at room temperature for 1 h. The fixed cells were collected by using a cell scraper, washed several times in 0.1 M phosphate buffer, and post fixed in 1% osmium tetroxide. Samples were dehydrated in ethanol (30%, 50%, 70%, 80%, 95%, and 100%), embedding in EMbed 812 Resin (Electron Microscopy Sciences, 14120) and polymerized. In all, 70–90-nm sections were cut on a Reichert-Jung ultramicrotome using a diamond knife (DiATOME). Sections were collected on copper grids, stained with UranyLess and lead citrate, and imaged with a Hitachi H-7000 TEM.

### In vivo mouse model ASO injection

Three-month-old MMTV-Neu-NDL mice were divided into three cohorts (7–12 mice each), and each mouse in the cohort received either scASO or *MaTAR25-*specific ASO1 or ASO2 via subcutaneous injection 50 mg/kg/day twice per week. The injections were carried out for a period of 7 weeks, upon which at least one tumor from most of the control mice reached 2 cm in size. During the course of treatment, tumors were measured twice per week. At the end of the treatment period, the animals were euthanized and the primary tumors, lungs, livers, spleens, and intestines were collected. Collected lungs were fixed in 4% paraformaldehyde and incubated in 20–30% sucrose solution overnight, then frozen in OCT solution (Tissue-Tek, 62550–12). The lung OCT blocks were cross-sectioned 2-mm apart, and the lung sections were embedded horizontally to obtain serial sections of the entire lung. Other tissues were cut into two pieces. One part of each issue was snap-frozen using liquid nitrogen for further RNA extraction. The remaining tissues were fixed in 4% paraformaldehyde and paraffin-embedded, then formalin-fixed, paraffin-embedded (FFPE) tissue blocks were sectioned. All sections were stained with H&E following standard protocol, and slides were scanned and analyzed using an Aperio ImageScope pathology slide viewing system. All samples were processed and stained at the Cold Spring Harbor Laboratory Histology Shared Resource. Slides were scanned and examined using Aperio ImageScope software.

### In vivo 4T1 cells injection

In all, 5–6-week-old female BALB/c mice and 4T1 cells (control and *MaTAR25* KO cells) expressing luciferase were used for 4T1 mammary fat pad and tail-vein injection experiments. For mammary fat pad injection, 1 × 10^5^ 4T1 control or *MaTAR25* KO cells were injected orthotopically into the mammary fat pad of female BALB/c mice. Mice were monitored and primary tumors were measured every week. Mice were sacrificed, and tumors were collected at day 28 to compare the tumor growth rate between 4T1 control groups and *MaTAR25* KO groups.

For tail-vein injection, female BALB/c mice were injected intravenously with 1 × 10^5^ 4T1 control or *MaTAR25* KO cells in the tail vein. Mice were monitored every week and sacrificed at day 21. The mouse lungs were collected and imaged, and lung metastatic nodules were counted to compare the metastatic ability between 4T1 control groups and *MaTAR25* KO groups.

### Quantification and statistical analysis

Statistics tests were performed and analyzed using Microsoft Excel and GraphPad Prism 7.0. *P* value was calculated by paired Student’s *t* test, two-tailed. Significance was defined as *P* < 0.05. *P* values of results are provided in the Source Data file.

### Outline of tools used in this study

UCSC genome browser (https://genome.ucsc.edu/)^81^

ensembl genome browser (http://useast.ensembl.org/index.html)

FANTOM5 (https://fantom.gsc.riken.jp/5/)

CPAT (http://lilab.research.bcm.edu/cpat/)^82^

CPC (http://cpc.gao-lab.org/)^83^

KM plotter (https://kmplot.com/analysis/)

TANRIC (https://ibl.mdanderson.org/tanric/_design/basic/query.html).

### Reporting summary

Further information on research design is available in the Nature Research Reporting Summary linked to this article.

## Supplementary information

Supplementary Information

Reporting Summary

Description of Additional Supplementary Files

Supplementary Data 1

Supplementary Data 2

Supplementary Data 3

Supplementary Data 4

Supplementary Data 5

Supplementary Data 6

Supplementary Data 7

Supplementary Data 8

Supplementary Data 9

Supplementary Data 10

Supplementary Movie 1

Supplementary Movie 2

## Data Availability

The accession number for the RNA-seq and ChIRP-seq data reported in this study is GEO: GSE142169. The dataset identifier for the mass spectrometry proteomics data deposited to the ProteomeXchange Consortium via the PRIDE partner repository is: PXD017398. All data are available from the corresponding author upon reasonable request. Source data are provided with this paper.

## Source data

Source Data
