# Supplementary material for: MaTAR25 lncRNA regulates the Tensin1 gene to impact breast cancer progression
Source: Nat Commun. 2020 Dec 22;11:6438. doi: 10.1038/s41467-020-20207-y (PMC7755919; doi:10.1038/s41467-020-20207-y)
Supplement: Supplementary file 1 — Supplementary Information [file 41467_2020_20207_MOESM1_ESM.pdf]

SUPPLEMENTARY INFORMATION

MaTAR25 **lncRNA regulates the** ***Tensin1*** **gene to impact breast cancer progression**

Kung-Chi Chang, Sarah D. Diermeier, Allen T. Yu, Lily D. Brine, Suzanne Russo, Sonam Bhatia, Habeeb Alsudani, Karen Kostroff, Tawfiqul Bhuiya, Edi Brogi, Darryl J. Pappin, C. Frank Bennett, Frank Rigo, and David L. Spector

Supp. Fig. 1

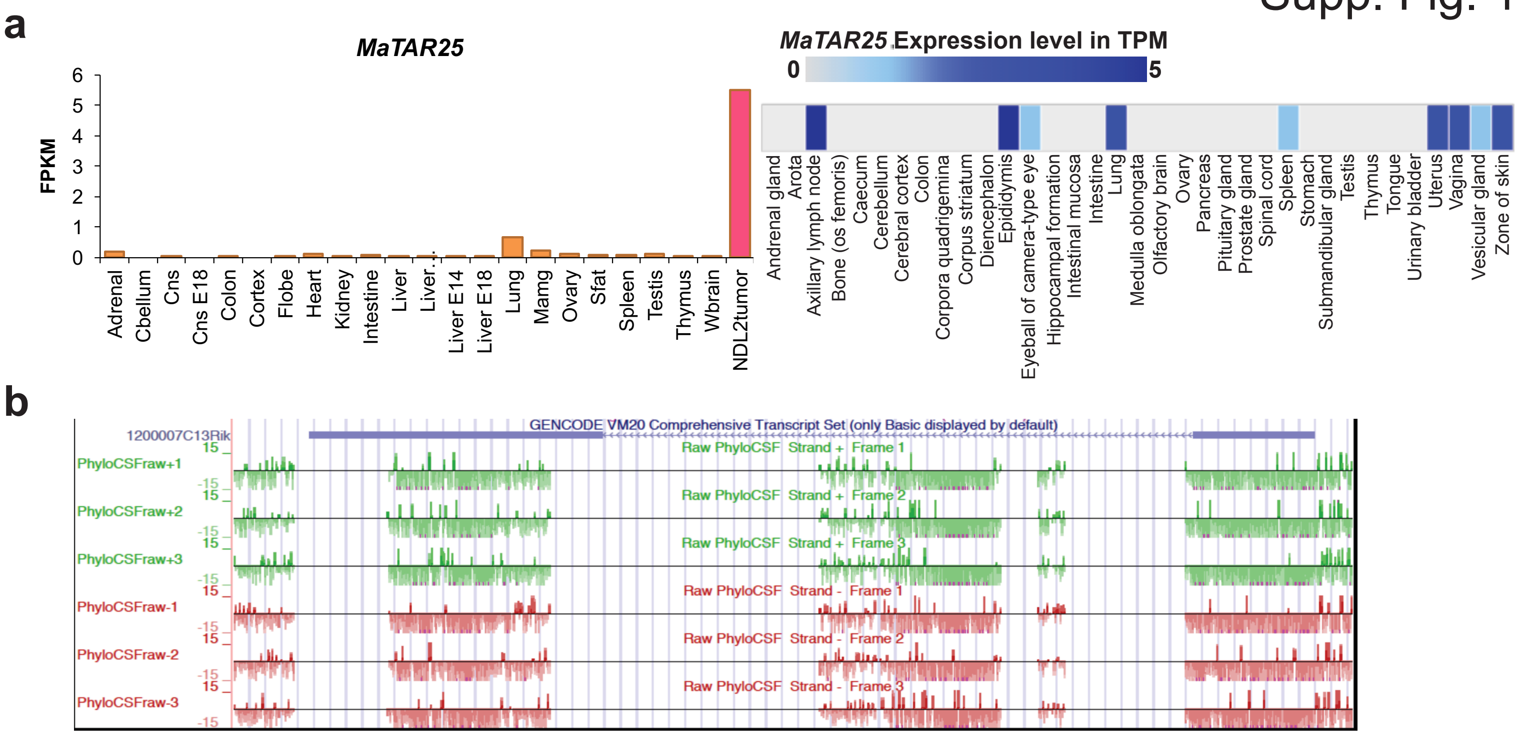

c

Result for species name : mm10 with job ID :1563826412

Data ID	Sequence Name	RNA Size	ORF Size	Ficket Score	Hexamer Score	Coding Probability	Coding Label
0	MATAR25	1978	369	0.8146	0.00331186776961	0.24503635507318	no

d

ID	C/NC	CODING POTENTIAL SCORE	EVIDENCE	UTR-DB HITs	RNA-DB HITs
MaTAR25	noncoding (weak)	-0.932493	detail	search	search

ORF INFORMATION

unreliable ORF

SOURCE	START	END	LENGTH	COVERAGE	SCORE	TYPE
ORF_FRAMEFINDER	1121	1487	367 (123AA)	18.50%	46.34	Full

PUTATIVE PEPTIDE

unreliable ORF

>MaTAR25 [framefinder (1120,1486) score=46.34 used=18.50% {forward,strict}]

MMTGDIPSLPWGEGGLWPGDKGSRPPTSGKRTDNRDSGQATSPGEGGHRMSVREDRDRGC
LMRPSARPTASLPGSRPLSCEVEPPFSLFYHPSSSQLAGVPSRCPLACGLGSEGKGTNPW
VN

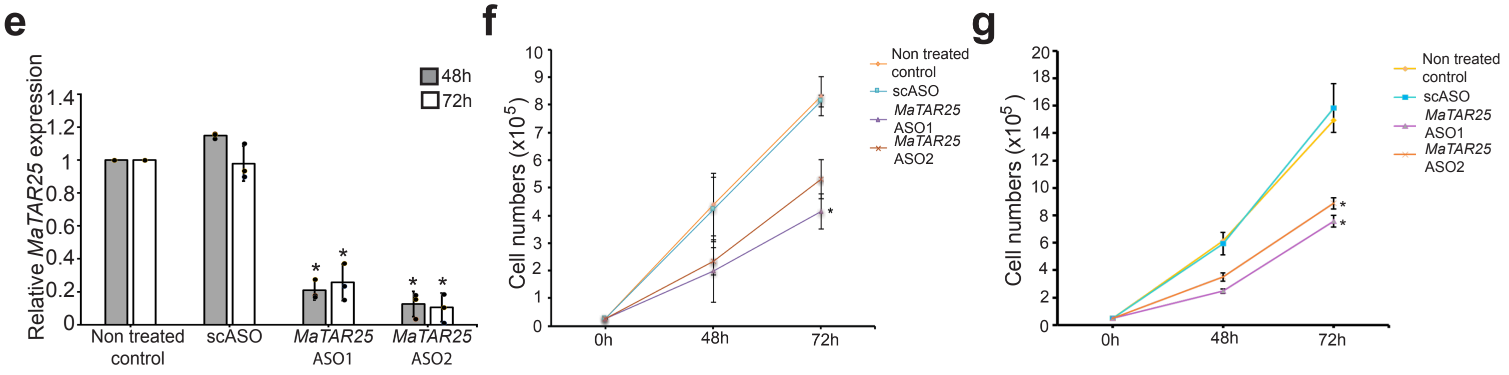

Supplementary Figure 1. Characterization of *Mammary Tumor Associated RNA 25* (*MaTAR25*).

Related to Figure 1.

a) Expression profile of *MaTAR25* in various mouse tissue types from ENCODE data sets compared to RNA-Seq results of mammary tumor from MMTV-Neu-NDL mice (ref. 30), and from FANTOM5 database (ref. 31).

b) Screen shot of *MaTAR25* genomic locus from UCSC genome browser showing PhyloCSFraw (ref. 81) scores of individual codons in each of three frames (+ and – strands; horizontal line is 0).

c) Protein coding potential analysis of *MaTAR25* RNA transcript using Coding-Potential Assessment Tool (CPAT) (ref. 82).

d) Assessing the protein coding potential of *MaTAR25* using Coding Potential Calculator (CPC) (ref. 83), and the predicted open reading frame (ORF) and putative peptide.

e) qRT-PCR showing the knockdown efficiency of 2 independent ASOs targeting *MaTAR25* in 4T1 cells after 48 hours and 72 hours of incubation compared to mock and scASO treated control cells. Data are presented as mean values ± SD (n=3 independent experiments). *$p < 0.05$ (paired student's t-test; two tailed).

f) 4T1 cells were seeded at the same cell density (2.5×10^4/well) in 12-well tissue culture plates at day 0. The mean cell numbers of three independent replicates of 4T1 mock treated control cells, 4T1 cells treated with scrambled ASO, 4T1 cells treated with 2 different *MaTAR25* ASOs is shown ± SD (n=3). *$p < 0.05$ (paired student's t-test; two tailed).

g) cNeu (MMTV-Neu-NDL) cells were seeded at the same cell density (5×10^4/well) in 12-well tissue culture plates at day 0 while ASOs were added into the culture medium and cell counting was performed at different time points to measure cell numbers. Data are presented as mean values ± SD (n=3 independent experiments). *$p < 0.05$ (student's t-test).

Supp. Fig. 2

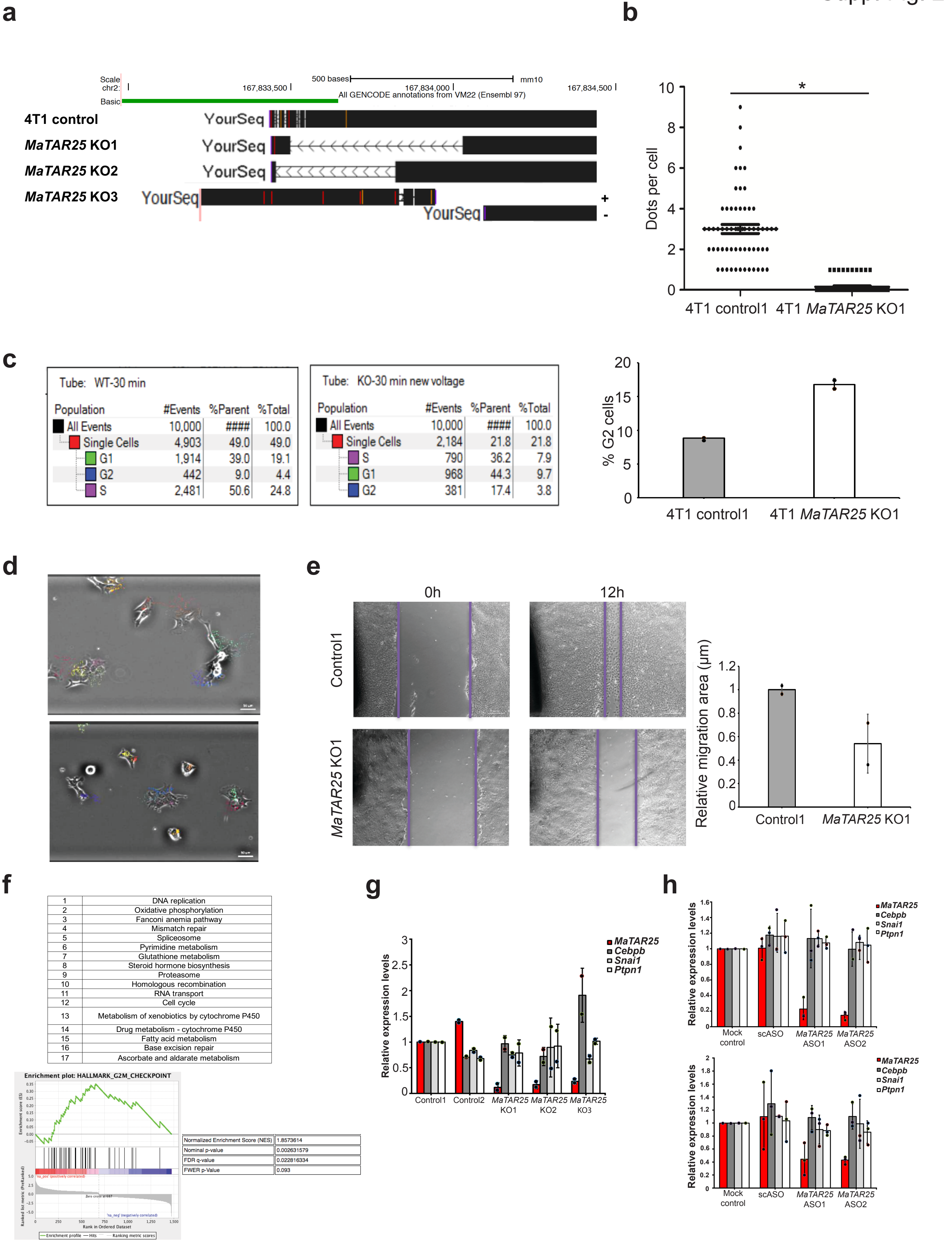

a
b
c
d
e
f
g
h
Scale
chr2:
500 bases
mm10
167,833,500
167,834,000
167,834,500
All GENCODE annotations from VM22 (Ensembl 97)
Basic
4T1 control
MaTAR25 KO1
MaTAR25 KO2
MaTAR25 KO3
YourSeq
+
-
Dots per cell
*
4T1 control1
4T1 MaTAR25 KO1
Tube: WT-30 min
Population #Events %Parent %Total
All Events 10,000 #### 100.0
Single Cells 4,903 49.0 49.0
G1 1,914 39.0 19.1
G2 442 9.0 4.4
S 2,481 50.6 24.8
Tube: KO-30 min new voltage
Population #Events %Parent %Total
All Events 10,000 #### 100.0
Single Cells 2,184 21.8 21.8
S 790 36.2 7.9
G1 968 44.3 9.7
G2 381 17.4 3.8
% G2 cells
0h
12h
Control1
MaTAR25 KO1
Relative migration area (µm)
1 DNA replication
2 Oxidative phosphorylation
3 Fanconi anemia pathway
4 Mismatch repair
5 Spliceosome
6 Pyrimidine metabolism
7 Glutathione metabolism
8 Steroid hormone biosynthesis
9 Proteasome
10 Homologous recombination
11 RNA transport
12 Cell cycle
13 Metabolism of xenobiotics by cytochrome P450
14 Drug metabolism - cytochrome P450
15 Fatty acid metabolism
16 Base excision repair
17 Ascorbate and aldarate metabolism
Enrichment plot: HALLMARK_G2M_CHECKPOINT
Normalized Enrichment Score (NES) 1.8573614
Nominal p-value 0.002631579
FDR q-value 0.022816334
FWER p-Value 0.093
Relative expression levels
MaTAR25
Cebpb
Snai1
Ptpn1
Control1
Control2
MaTAR25 KO1
MaTAR25 KO2
MaTAR25 KO3
Mock control
scASO
MaTAR25 ASO1
MaTAR25 ASO2

Supplementary Figure 2. *MaTAR25* knockout affects 4T1 cell cycle progression, and migration as well as related regulatory pathways *in vitro.*

Related to Figure 2.

a) A snapshot of BLAT results showing the CRISPR-Cas9 mediated editing events of 3 different 4T1 *MaTAR25* knockout (KO) clones over the promoter region of the *MaTAR25* gene locus by aligning Sanger sequencing results on the UCSC genome browser.
b) Quantitation of the number of *MaTAR25* smRNA-FISH foci in individual 4T1 control and *MaTAR25* KO cells. 67 cells were counted in control group and 70 *MaTAR25* KO cells were counted. The data are presented as mean values ± SD. **p* < 0.05 (paired student's t-test; two tailed).
c) BrdU incorporation cell cycle analysis using flow cytometry to compare the percentage of cells in each cell cycle stage between 4T1 control and *MaTAR25* KO cells. The percentage of cells in G2 phase in 4T1 control1 vs *MaTAR25* KO1 cells ± SD (n=2 independent experiments).
d) Live cell images from tracking 4T1 control cells and *MaTAR25* KO cells over time. More than 3 independent experiments were performed and representative images are shown.
e) Scratch wound healing assay showing the difference in migration ability between 4T1 control1 vs *MaTAR25* KO1 cells. The wound line was created by a microtip, then cells were rinsed with PBS twice before adding new medium and incubated for 12 hours. The migration areas in each group were measured using ImageJ and the result is shown ± SD (n=2 independent experiments).
f) Pathway analysis showing affected pathways in *MaTAR25* KO cells when comparing differentially expressed genes identified by RNA-Seq analysis from *MaTAR25* KO cells compared to 4T1 control cells.
g) qRT-PCR analysis showing the expression levels of *MaTAR25* and its three neighboring protein coding genes (*Snai1, Cebpb, and Ptpn1*) between 4T1 control clones and *MaTAR25* KO clones. The result is shown ± SD (n=2 independent experiments).
h) qRT-PCR showing the expression levels of *MaTAR25* and its three neighboring protein coding genes (*Snai1, Cebpb, and Ptpn1*) in 4T1 and cNeu cells after 48 hours KD using 2 independent ASOs targeting *MaTAR25* compared to mock or scASO treated control cells. Data are presented as mean values ± SD (n=3 independent experiments). **p*<0.05 (paired student's t-test; two tailed).

Supp. Fig. 3

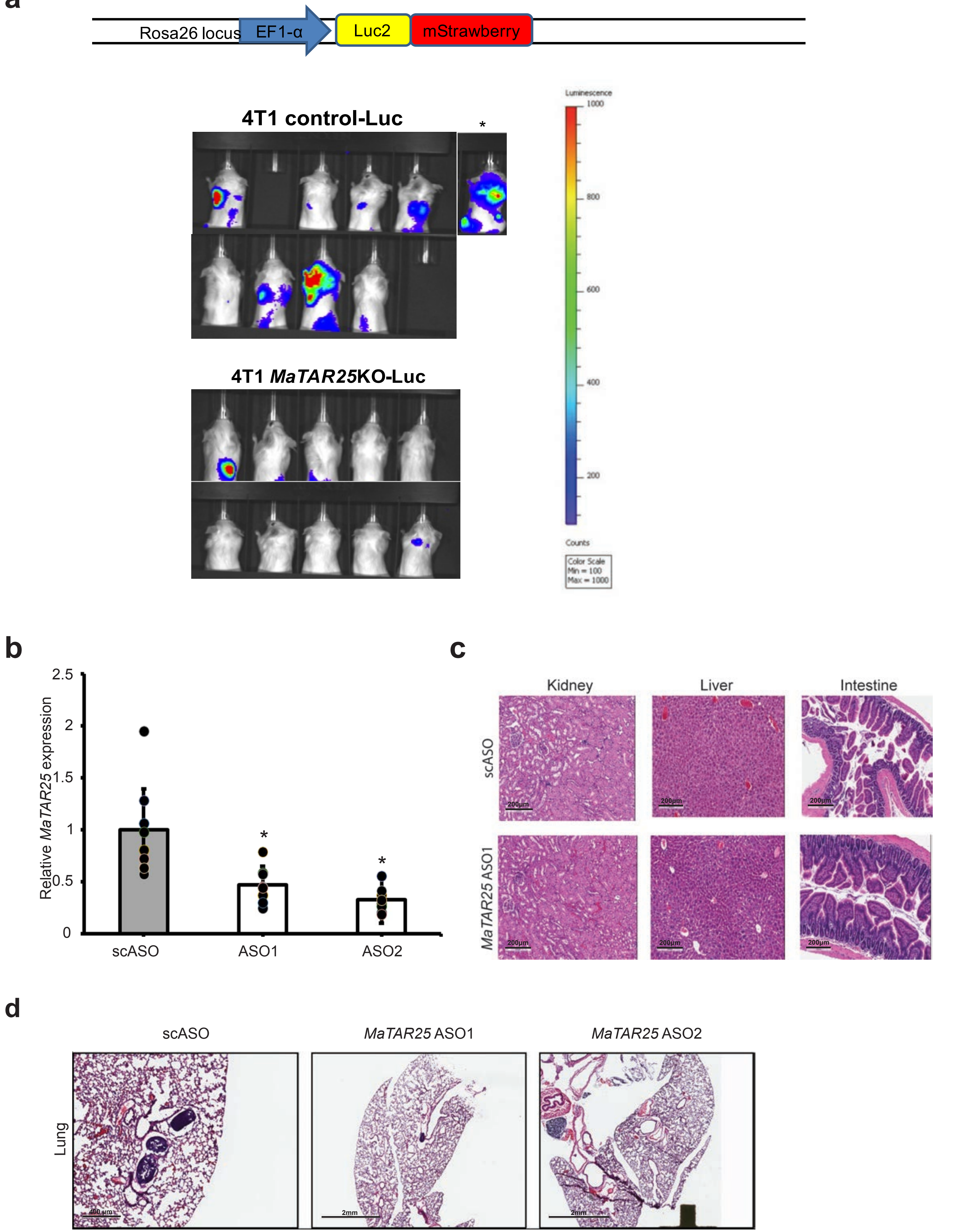
a
Rosa26 locus
EF1-α
Luc2
mStrawberry
4T1 control-Luc
*
4T1 *MaTAR25*KO-Luc
Luminescence
1000
800
600
400
200
Counts
Color Scale
Min = 100
Max = 1000
b
Relative *MaTAR25* expression
2.5
2
1.5
1
0.5
0
*
*
scASO
ASO1
ASO2
c
Kidney
Liver
Intestine
scASO
MaTAR25 ASO1
200μm
d
scASO
MaTAR25 ASO1
MaTAR25 ASO2
Lung
400 μm
2mm
2mm

Supplementary Figure 3. *MaTAR25* knockout impairs lung metastasis *in vivo.* Related to Figure 3.

a) Schematic representation of the plasmid construct targeted into the Rosa26 locus for positive clone selection and *in vivo* bioluminescence imaging of luciferase labeled 4T1 control and *MaTAR25* KO cells. The *in vivo* bioluminescence images were acquired at day 21 from female BALB/c mice tail vein injected with 4T1 control or *MaTAR25* KO cells.
b) qRT-PCR analysis showing the *in vivo* knockdown efficiency of 2 independent ASOs targeting *MaTAR25* in tumors collected from MMTV-Neu-NDL mice compared to tumors from scASO injected mice. Data are presented as mean values ± SD (n=8 tumor samples each group). *$p < 0.05$ (paired student's t-test; two tailed).
c) Hematoxylin and eosin (H&E) stained tissue images (kidney, liver and intestine) showing no histological phenotypes between scASO treated and *MaTAR25* ASO treated groups. Normal tissues are not impacted by *MaTAR25* knockdown. 2 different tissue samples in each group were examined and representative images are shown.
d) Representative hematoxylin and eosin (H&E) stained lung images showing reduced micro-metastatic nodules in lung tissue from mice treated with each of two *MaTAR25* ASOs vs scASO control. 7 different lung samples in each group were examined and representative images are shown.

Supp. Fig. 4

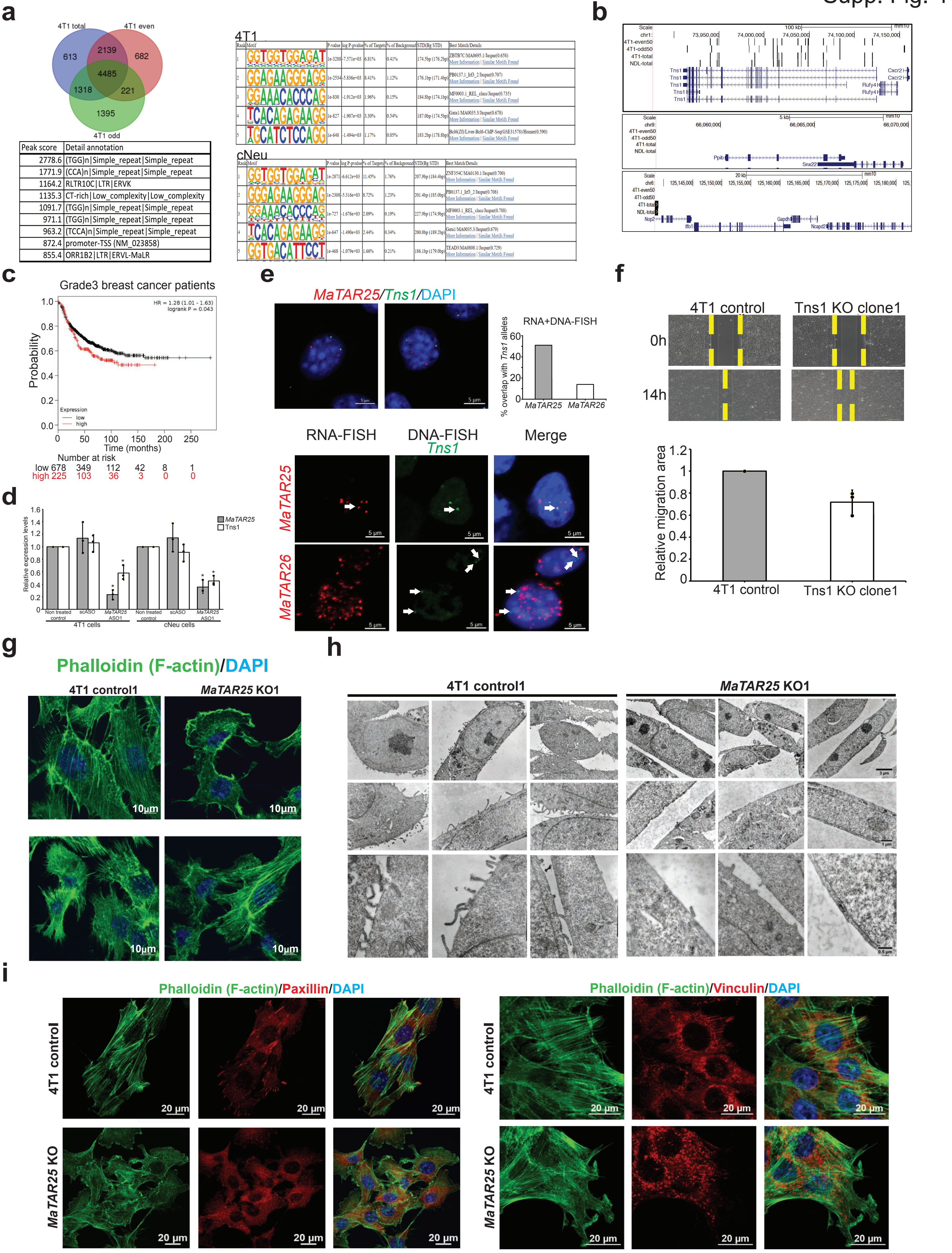

Peak score	Detail annotation
2778.6	(TGG)n|Simple_repeat|Simple_repeat
1771.9	(CCA)n|Simple_repeat|Simple_repeat
1164.2	RLTR10C|LTR|ERVK
1135.3	CT-rich|Low_complexity|Low_complexity
1091.7	(TGG)n|Simple_repeat|Simple_repeat
971.1	(TGG)n|Simple_repeat|Simple_repeat
963.2	(TCCA)n|Simple_repeat|Simple_repeat
872.4	promoter-TSS (NM_023858)
855.4	ORR1B2|LTR|ERVL-MaLR

Supplementary Figure 4. *Tns1* is the key downstream target of *MaTAR25* for regulating tumor cell progression and migration.

Related to Figure 4.

a) Venn diagram showing the overlap of genomic target sites from ChIRP-Seq data using *MaTAR25* total oligo pool (14 oligos), *MaTAR25* odd oligo pool (7 oligos), or *MaTAR25* even oligo pool (7 oligos) for RNA pull-down in 4T1 cells. The number of target sites is indicated by the overlap between the circles (top panel).
   Top genome ontology enrichment regions from analyzing the sequence of overlapped target sites are listed in the lower panel. An enriched transcription factor motif sequence was identified from 4T1 and cNeu cells *MaTAR25* ChIRP-seq data using HOMER (ref. 69) (right panel).

b) UCSC genome browser snapshot showing peaks of *MaTAR25* ChIRP-Seq data over *Tns1* vs two non-target genes (*Ppib, Gapdh*) using *MaTAR25* odd oligo pool (7 oligos), *MaTAR25* even oligo pool (7 oligos), or *MaTAR25* total oligo pool (14 oligos) for RNA pull-down in 4T1 cells, and *MaTAR25* total oligo pool (14 oligos) for RNA pull-down in MMTV-NDL cells.

c) Kaplan Meier-plotter analysis by KM plotter (ref. 38) showing the correlation of *Tns1* expression levels (lower and upper quartiles were computed and indicated as low expression and high expression groups) with survival of patients with Grade 3 breast tumors.

d) qRT-PCR showing the knockdown efficiency of *MaTAR25 ASO1* and the changes of Tns1 expression in 4T1 cells and cNeu after 72 hours of incubation compared to mock and scASO treated control cells. Data are presented as mean values ± SD (n=3 independent experiments). *$p < 0.05$ (student's t-test).

e) Representative images of double DNA-FISH detecting *MaTAR25* (red) and *Tns1* (green) gene loci in 4T1 cells (upper panel). Representative images of smRNA-FISH of *MaTAR25* RNA transcripts (red) and DNA-FISH detecting *Tns1* gene loci (green) within the same cell to assess the potential interaction between *MaTAR25* RNA transcripts and *Tns1* gene loci in 4T1 cells. lncRNA *MaTAR26* (red) was used as a control (lower panel). Total 60 cells in each sample set were imaged and counted for quantification of % overlap smRNA signals with *Tns1* alleles (upper panel). Scale bars are 5 μm.

f) Scratch wound healing assay showing the difference in migration ability between 4T1 control and *Tns1* KO cells. The migration areas in each group were measured by ImageJ and the result is shown ± SD (n=2 independent experiments).

g) Images showing the changes of F-actin bundles in 4T1 control cells, 4T1 *MaTAR25* KO cells, 4T1 *MaTAR25* KO cells with ectopic expression of *MaTAR25*, and 4T1 *MaTAR25* KO cells with ectopic expression of Tns1. Cells were stained with Alexa Fluor 488 phalloidin conjugate (green), and images were acquired using a Zeiss 710 confocal microscope. More than 3 independent experiments were performed and the representative images are shown. Scale bars are 10 μm.

h) Transmission electron microscopy (TEM) images showing the significant reduction in microvilli upon *MaTAR25* KO. 2 different experiments were examined and the

representative images are shown. Shared scale bars are indicated in the right panels (3, 1, and 0.5 μm).

i) Images showing the changes in the localization of focal adhesion complex proteins in 4T1 control and 4T1 *MaTAR25* KO cells by immunofluorescence (IF) labeling (red) of paxillin (upper panel) and vinculin (lower panel). Cells were co-stained with Alexa Fluor 488 phalloidin conjugate (green). 3 independent experiments were performed and representative images are shown. Scale bars are 20 μm.

Supp. Fig. 5

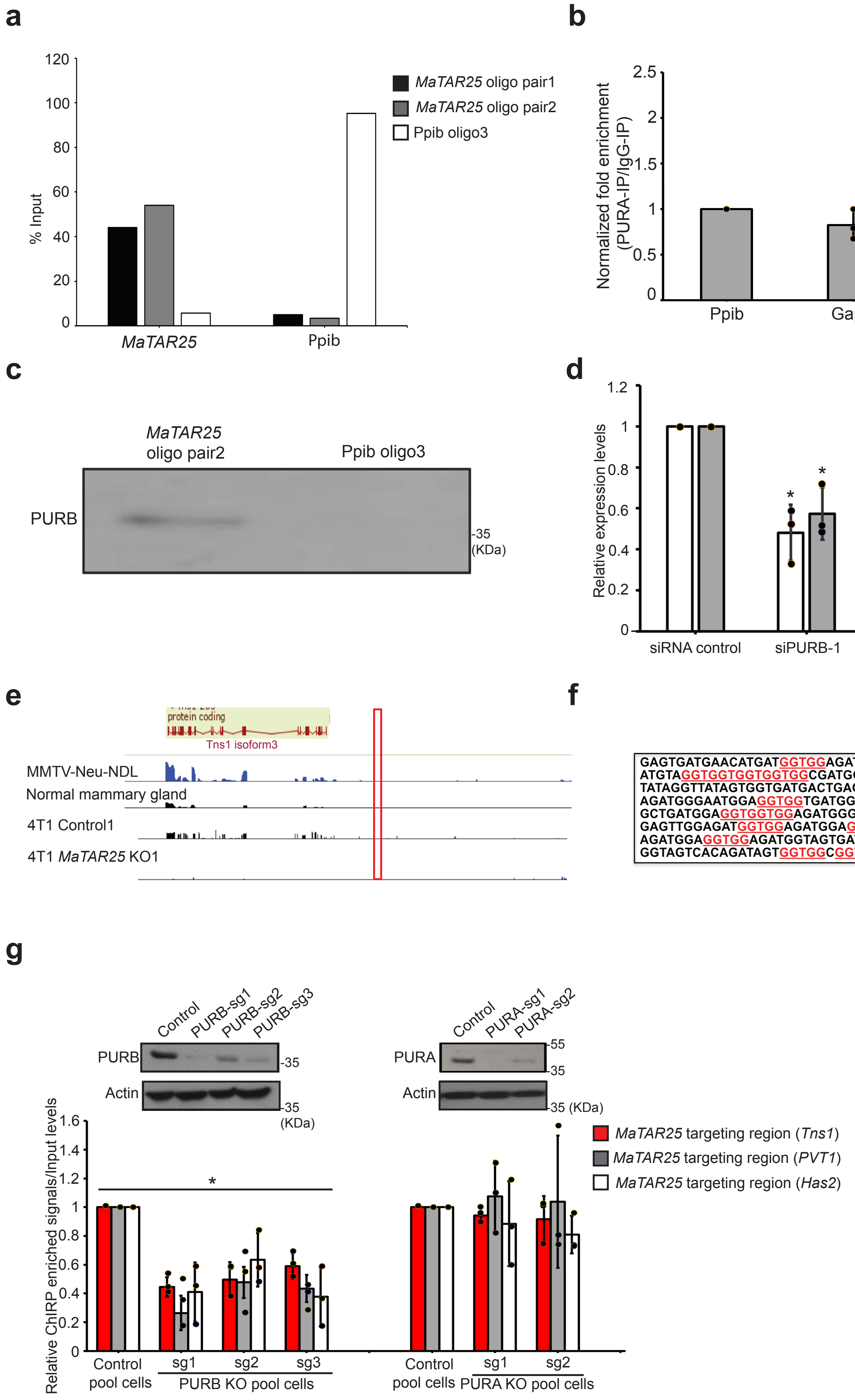

a
% Input
120
100
80
60
40
20
0
MaTAR25 oligo pair1
MaTAR25 oligo pair2
Ppib oligo3
MaTAR25
Ppib
b
Normalized fold enrichment (PURA-IP/IgG-IP)
2.5
2
1.5
1
0.5
0
Ppib
Gapdh
MaTAR25
c
MaTAR25 oligo pair2
Ppib oligo3
PURB
-35 (KDa)
d
Relative expression levels
1.2
1
0.8
0.6
0.4
0.2
0
PURB
Tns1
siRNA control
siPURB-1
siPURB-2
e
protein coding
Tns1 isoform3
MMTV-Neu-NDL
Normal mammary gland
4T1 Control1
4T1 MaTAR25 KO1
f
GAGTGATGAACATGATGGTGGAGATGATGGCG
ATGTAGGTGGTGGTGGTGGCGATGGAGGAGA
TATAGGTTATAGTGGTGATGACTGAGATGATGG
AGATGGGAATGGAGGTGGTGATGGAGATGAT
GCTGATGGAGGTGGTGGAGATGGGAATGGTA
GAGTTGGAGATGGTGGAGATGGAGGTGGTGA
AGATGGAGGTGGAGATGGTAGTGATGAAGAT
GGTAGTCACAGATAGTGGTGGCGGTGGTAATG
g
Control
PURB-sg1
PURB-sg2
PURB-sg3
PURB
-35
Actin
-35 (KDa)
Control
PURA-sg1
PURA-sg2
PURA
-55
-35
Actin
-35 (KDa)
Relative ChIRP enriched signals/Input levels
1.6
1.4
1.2
1
0.8
0.6
0.4
0.2
0
MaTAR25 targeting region (Tns1)
MaTAR25 targeting region (PVT1)
MaTAR25 targeting region (Has2)
Control pool cells
sg1
sg2
sg3
PURB KO pool cells
Control pool cells
sg1
sg2
PURA KO pool cells

Supplementary Figure 5. *MaTAR25* interacts with PURB to carry out its function. Related to Figure 5.

a) *MaTAR25* or PPIB (control) RNA transcripts were assessed by qRT-PCR following the pull-down of *MaTAR2*5 or *PPIB* in 4T1 cells.
b) *MaTAR25*, PPIB, and GAPDH transcripts were assessed by qRT-PCR in endogenous PURA, or IgG (negative control) immunoprecipitates from 4T1 cells. Fold enrichment of PURA associated RNA signal over IgG signal is calculated and data are presented as mean values ± SD (n=3 independent experiments).
c) Immunoblot analysis of PURB following the pull-down of *MaTAR25* or PPIB in MMTV-Neu-NDL primary cells. 2 independent experiments were performed and a representative blot image is shown.
d) qRT-PCR analysis of *Tns1* expression in 4T1 cells following treatment for 48 hours with three independent siRNAs to knockdown PURB, compared to negative control cells. Data are presented as mean values ± SD (n=3 independent experiments). $*p < 0.05$ (paired student's t-test; two tailed).
e) Ensemble genome browser snapshot showing isoform3 of *Tns1* and UCSC genome browser snapshot showing the expression levels of *Tns1* between MMTV-Neu-NDL, normal mammary gland RNA-Seq results (ref. 30), 4T1 control1 and 4T1 *MaTAR25* KO1 RNA-Seq results. The red square highlights the *MaTAR25* targeting region over Tns1 isoform 3 promoter from ChIRP-Seq data sets.
f) The DNA targeting sequence of *Tns1* DNA recognized by *MaTAR25* by ChIRP-Seq analysis. The PURB binding sequence motif (GGTGG) is highlighted.
g) *MaTAR25* oligos (even 7) ChIRP-PCR analysis of *MaTAR25* targeting regions over three downstream genes (*Tns1, PVT1, Has2*) using 4T1 control pool cells, PURB KO pool cells (sg1-3), and PURA KO pool cells (sg1-2). Data are presented as mean values ± SD (n=3 independent experiments). $*p < 0.05$ (paired student's t-test; two tailed).

Supp. Fig. 6

a

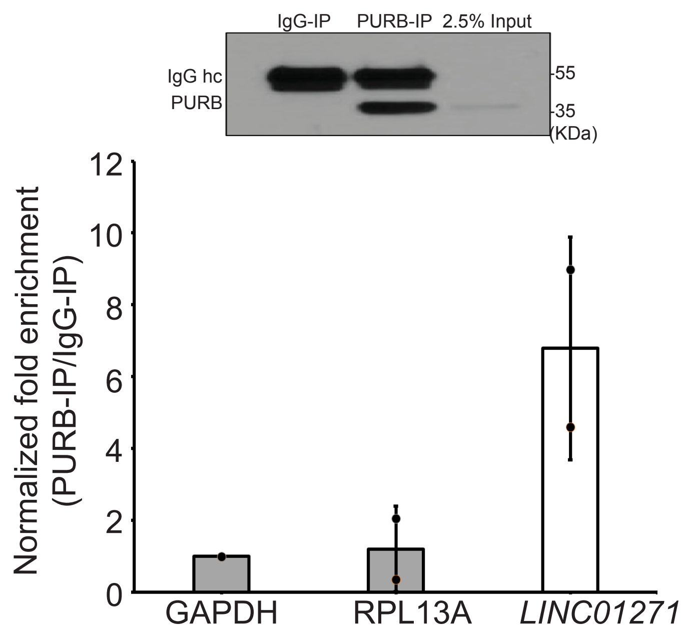

b

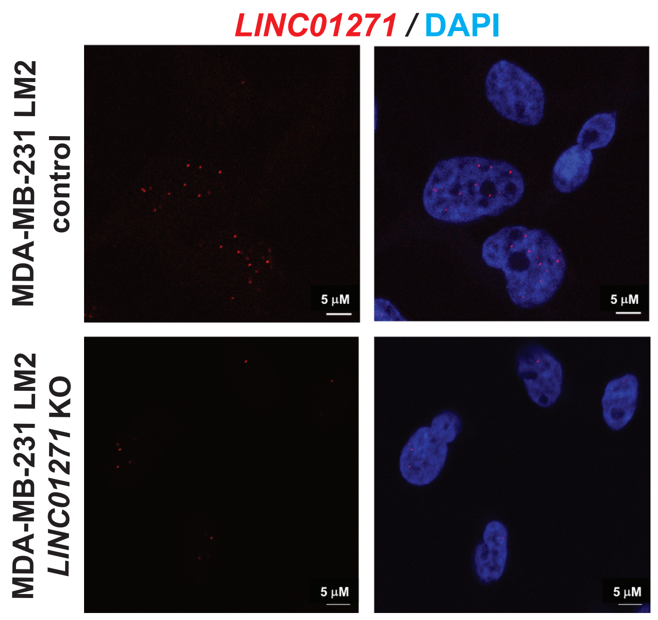

c

Positive correclation
DNA REPLICATION
MITOTIC M M G1 PHASES
CELL CYCLE MITOTIC
M G1 TRANSITION
SYNTHESIS OF DNA
CELL CYCLE
S PHASE

d

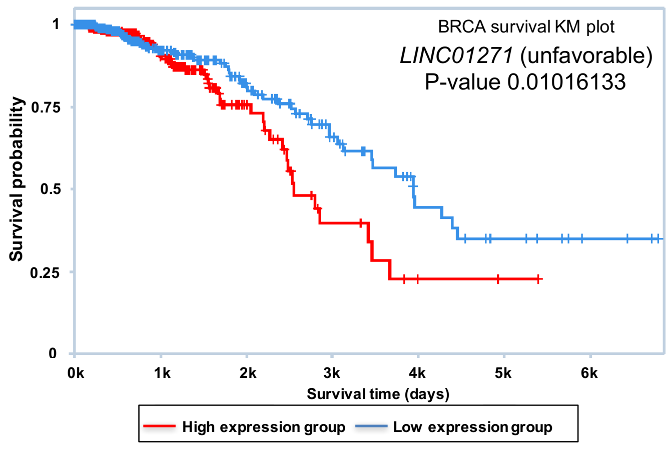

e

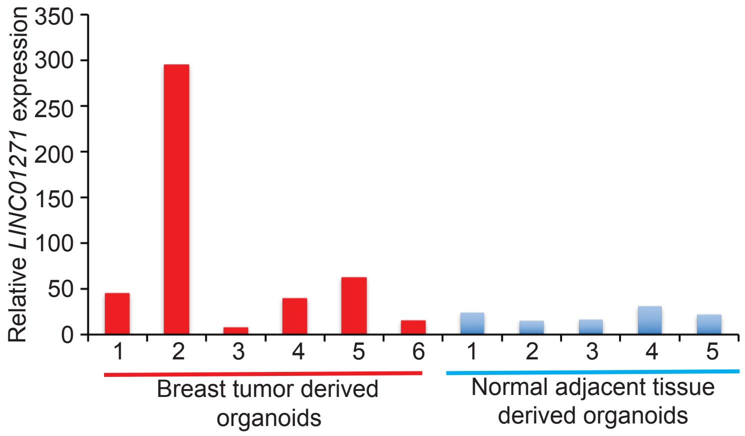

Supplementary Figure 6. *LINC01271* is the human ortholog of *MaTAR25*. Related to Figure 6.

a) RNA immunoprecipitation (RIP) and qRT-PCR using PURB antibody to identify the specific interaction between PURB and *LINC01271* RNA transcripts in MDA-MB-231 LM2 cells. Rabbit IgG antibody was used as a negative control. qRT-PCR detecting *Gapdh* and *Rpl13a* RNA transcripts were used as non-specific RNA transcript binding for normalization (n=2 independent experiments).
b) Images of smRNA-FISH detecting *LINC01271* RNA transcripts (red) in MDA-MB-231 LM2 cells to assess the localization of *LINC01271* in control and *LINC01271* KO cells. 2 independent experiments were performed and representative images are shown. Scale bars are 5 μm.
c) Identification of the positive correlation pathways with *LINC01271* expression by reactome analysis based on the Breast Cancer TCGA data.
d) Kaplan Meier-plotter analysis by TANRIC (ref. 45) showing the correlation of *LINC01271* expression level (lower and upper quartiles from 837 tumor samples were computed and indicated as low expression and high expression groups) on survival of breast cancer patients. *p*-value was calculated from the univariate cox proportional hazards model with a log-rank test.
e) Comparison of the expression level of *LINC01271* within breast tumor organoids derived from samples of luminal subtype breast cancer patient vs normal breast organoids derived from adjacent breast tissues detected by qRT-PCR.

Supp. Fig. 7

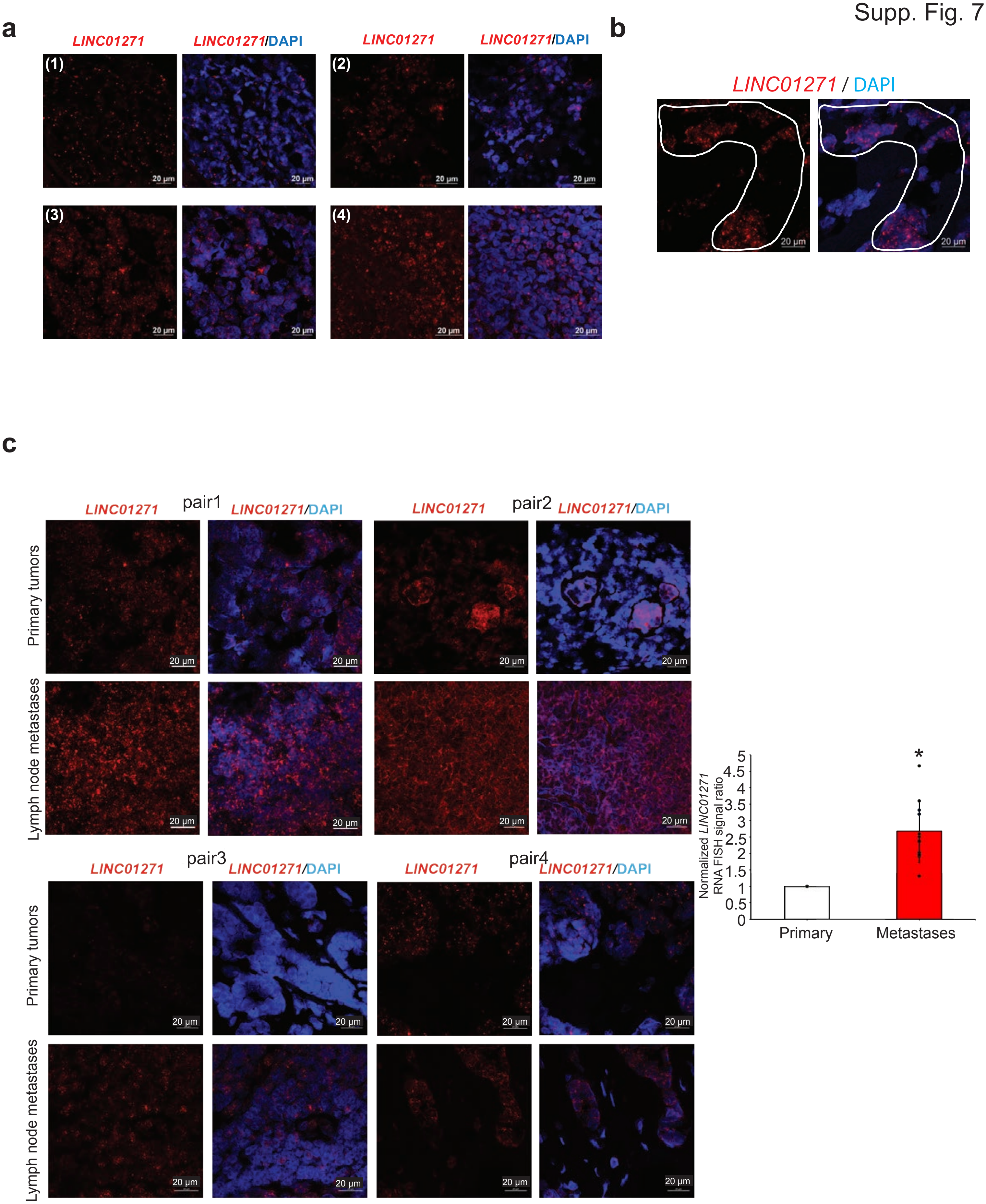
a
LINC01271
LINC01271/DAPI
(1)
(2)
(3)
(4)
20 µm
b
LINC01271 / DAPI
c
pair1
pair2
pair3
pair4
Primary tumors
Lymph node metastases
Normalized LINC01271 RNA FISH signal ratio
0
0.5
1
1.5
2
2.5
3
3.5
4
4.5
5
*
Primary
Metastases

Supplementary Figure 7. smRNA-FISH examining *LINC01271* expression in patient breast tumor samples.

Related to Figure 7.

a) smRNA-FISH images showing the expression of *LINC01271* (red) in patient breast tumor sections from early stage (1) (2) and late stage (3) (4) breast tumor sections. 6 random areas on each slide were examined and representative images are shown. Scale bars are 20 μm.

b) Representative smRNA-FISH images showing the clonal/regional expression pattern of *LINC01271* RNA transcripts (red) within breast tumor patient samples. Regions containing cells with higher expression level of *LINC01271* are outlined. 6 random areas each slide were compared and representative images are shown. Scale bars are 20 μm.

c) smRNA-FISH images showing the expression pattern of *LINC01271* (red) within luminal subtype breast cancer primary tumors and lymph node metastases sections from the same patients. Scale bars are 20 μm. Total 11 patient sample sets (primary and metastatic) were imaged and counted for quantification. Data are presented as mean values ± SD * $p < 0.05$ (paired student's t-test; two tailed).
