# Supplementary material for: MaTAR25 lncRNA regulates the Tensin1 gene to impact breast cancer progression
Source: Nat Commun. 2020 Dec 22;11:6438. doi: 10.1038/s41467-020-20207-y (PMC7755919; doi:10.1038/s41467-020-20207-y)
Supplement: Supplementary file 2 — Reporting Summary [file 41467_2020_20207_MOESM2_ESM.pdf]

nature research

Corresponding author(s): David L. Spector

Last updated by author(s): Nov 18, 2020

Reporting Summary

Nature Research wishes to improve the reproducibility of the work that we publish. This form provides structure for consistency and transparency in reporting. For further information on Nature Research policies, see Authors & Referees and the Editorial Policy Checklist.

Statistics

For all statistical analyses, confirm that the following items are present in the figure legend, table legend, main text, or Methods section.

n/a	Confirmed	
☐	☒	The exact sample size (*n*) for each experimental group/condition, given as a discrete number and unit of measurement
☐	☒	A statement on whether measurements were taken from distinct samples or whether the same sample was measured repeatedly
☐	☒	The statistical test(s) used AND whether they are one- or two-sided<br>*Only common tests should be described solely by name; describe more complex techniques in the Methods section.*
☒	☐	A description of all covariates tested
☒	☐	A description of any assumptions or corrections, such as tests of normality and adjustment for multiple comparisons
☐	☒	A full description of the statistical parameters including central tendency (e.g. means) or other basic estimates (e.g. regression coefficient) AND variation (e.g. standard deviation) or associated estimates of uncertainty (e.g. confidence intervals)
☐	☒	For null hypothesis testing, the test statistic (e.g. *F*, *t*, *r*) with confidence intervals, effect sizes, degrees of freedom and *P* value noted<br>*Give P values as exact values whenever suitable.*
☒	☐	For Bayesian analysis, information on the choice of priors and Markov chain Monte Carlo settings
☒	☐	For hierarchical and complex designs, identification of the appropriate level for tests and full reporting of outcomes
☒	☐	Estimates of effect sizes (e.g. Cohen's *d*, Pearson's *r*), indicating how they were calculated

Our web collection on statistics for biologists contains articles on many of the points above.

Software and code

Policy information about availability of computer code

Data collection	We have not developed any software or code in this project.
Data analysis	R, https://www.r-project.org/<br>FACSDiva 8.0.1, Becton Dickinson<br>FlowJo v10, FLOWJO, LLC<br>ImageJ Fiji, NIH<br>CellTracker ver.1.1, CellTracker<br>GraphPad Prism 7.0, GraphPas Software<br>Aperio ImageScope 12.3, LEICA<br>ZEN microscope software, ZEISS<br><br>Oneline tools:<br>UCSC genome browser (https://genome.ucsc.edu/)<br>ensembl genome browser (http://useast.ensembl.org/index.html)<br>FANTOM5 (https://fantom.gsc.riken.jp/5/)<br>CPAT (http://lilab.research.bcm.edu/cpat/)<br>CPC (http://cpc.gao-lab.org/)<br>KM plotter (https://kmplot.com/analysis/)<br>TANRIC (https://ibl.mdanderson.org/tanric/_design/basic/query.html)

For manuscripts utilizing custom algorithms or software that are central to the research but not yet described in published literature, software must be made available to editors/reviewers. We strongly encourage code deposition in a community repository (e.g. GitHub). See the Nature Research guidelines for submitting code & software for further information.

Data

Policy information about availability of data

All manuscripts must include a data availability statement. This statement should provide the following information, where applicable:

- Accession codes, unique identifiers, or web links for publicly available datasets
- A list of figures that have associated raw data
- A description of any restrictions on data availability

The accession number for the RNA-seq and ChIRP-seq data reported in this study is GEO: GSE142169 (https://www.ncbi.nlm.nih.gov/geo/query/acc.cgi?acc=GSE142169)
The dataset identifier for the mass spectrometry proteomics data deposited to the ProteomeXchange Consortium via the PRIDE partner repository is: PXD017398 (https://www.ebi.ac.uk/pride/)
Source data for figures shown in this study are available upon request if not available in Supplementary Data and in the attached source data file. The source data underlying Figs. 2a-f, 3a-d, 4a, b, d-g, 5b-e, 6b-e, 7a and Supplementary Figs. 1e-g, 2b, c, e, g, h 3b, 4d-f, 5a-d, g, 6a, e, 7c are provided as a Source Data file.

Field-specific reporting

Please select the one below that is the best fit for your research. If you are not sure, read the appropriate sections before making your selection.

☒ Life sciences ☐ Behavioural & social sciences ☐ Ecological, evolutionary & environmental sciences

For a reference copy of the document with all sections, see nature.com/documents/nr-reporting-summary-flat.pdf

Life sciences study design

All studies must disclose on these points even when the disclosure is negative.

Sample size	Sample size of mouse model experiments were determined through guidelines from IACUC and previous studies from other groups. (https://www.bu.edu/researchsupport/compliance/animal-care/working-with-animals/research/sample-size-calculations-iacuc/) (https://pubmed.ncbi.nlm.nih.gov/24382818/) (https://www.ncbi.nlm.nih.gov/pmc/articles/PMC4701977/)More details are described in the methods, figure legends and supplementary figure legends.
Data exclusions	Data has not been excluded.
Replication	The experiments were performed in duplicate or triplicate as indicated in Figure legends to verify that the results. These data can be reproducible successfully.
Randomization	Female mice for the in vivo experiments were randomized by weight and age into different experimental groups. Since this study is focused on breast cancer only female mice were used. For other in vitro/ non-clinical experiments, no randomization was necessary since the experiments were duplicate and triplicate to verify the results.
Blinding	Tumors were measured without prior knowledge of MaTAR25 ASO treatment or scASO treatment.<br>For other in vitro experiments, no blinding was necessary since the experiments were duplicate and triplicate to verify the results.

Reporting for specific materials, systems and methods

We require information from authors about some types of materials, experimental systems and methods used in many studies. Here, indicate whether each material, system or method listed is relevant to your study. If you are not sure if a list item applies to your research, read the appropriate section before selecting a response.

Materials & experimental systems

n/a	Involved in the study	
☐	☒	Antibodies
☐	☒	Eukaryotic cell lines
☒	☐	Palaeontology
☐	☒	Animals and other organisms
☒	☐	Human research participants
☒	☐	Clinical data

Methods

n/a	Involved in the study	
☒	☐	ChIP-seq
☐	☒	Flow cytometry
☒	☐	MRI-based neuroimaging

Antibodies

Antibodies used	Rabbit polyclonal anti-Tensin-1 (for western) Signalway Antibody LLC Cat# 45400; RRID: N/A<br>Rabbit polyclonal anti-PURA (for western) abcam Cat# ab79936; RRID: AB_2253242

	Rabbit polyclonal anti-PURA (for RIP) abcam Cat# ab125200, RRID:AB_10973560<br>Rabbit polyclonal anti-PURB (for western, RIP, and ChIP) Proteintech Cat# 18128-1-AP; RRID: N/A<br>Rabbit monoclonal [Y113] anti-Paxillin (for IF) abcam Cat# ab32084; RRID: AB_779033<br>Rabbit monoclonal [EPR8185] anti-Vinculin (for IF) abcam Cat# ab129002; RRID: AB_11144129<br>Mouse monoclonal Anti-β-Actin (for western) Sigma-Aldrich Cat# A5441; AB_476744<br>Rabbit IgG Isotype Control ThermoFisher Catalog # 10500C; RRID: AB_2532981<br>Goat anti-Rabbit IgG (H+L) Cross-Adsorbed Secondary Antibody, Alexa Fluor 594 Invitrogen Catalog # A-11012
Validation	All antibodies were used ad per manufacturer's recommendations. And the dilution ratios are mentioned in methods and source data file.<br><br>Tensin-1 (https://www.sabbiotech.com/g-19435-Tensin-1-Antibody-45400.html)<br>PURA (western blot) (https://www.abcam.com/pura-antibody-ab79936.html)<br>PURA (RIP) (https://www.abcam.com/products?keywords=ab125200%2C)<br>PURB (https://www.ptglab.com/products/PURB-Antibody-18128-1-AP.htm)<br>Paxillin (https://www.abcam.com/paxillin-antibody-y113-ab32084.html)<br>Vinculin (https://www.abcam.com/vinculin-antibody-epr8185-ab129002.html)<br>β-Actin (https://www.sigmaaldrich.com/catalog/product/sigma/a5441?lang=en&region=US)<br>Rabbit IgG (https://www.thermofisher.com/antibody/product/Rabbit-IgG-Isotype-Control/10500C)<br>Goat anti-Rabbit IgG Alexa-594 (https://www.thermofisher.com/antibody/product/Goat-anti-Rabbit-IgG-H-L-Cross-Adsorbed-Secondary-Antibody-Polyclonal/A-11012)

Eukaryotic cell lines

Policy information about cell lines

Cell line source(s)	Murine mammary tumor cell line 4T1 (Fred Miller Lab)<br>Murine mammary tumor cell line NF639 (ATCC Cat# CRL-3090)<br>Human breast cancer cell line MDA-MB-231 LM2 (Joan Massagué Lab)
Authentication	4T1 cells were obtained from Fred R. Miller (Wayne State University), but not authenticated.
Mycoplasma contamination	Cold Spring Harbor Laboratory (CSHL) has a centralized cell culture facility and all cells are tested for mycoplasma on a routine basis. All cell lines used in this studies were Mycoplasma free.
Commonly misidentified lines<br>(See ICLAC register)	We have not used any commonly misidentified cell lines in our study.

Animals and other organisms

Policy information about studies involving animals; ARRIVE guidelines recommended for reporting animal research

Laboratory animals	3-4 month old FVB female NeuNDL mice, and 3 month old Balb/c female mice were used in this study. Details are described in the manuscript methods section.
Wild animals	Wild animals were not used in this study.
Field-collected samples	Field-collected samples were not used in this study.
Ethics oversight	Our animal protocol (18-15-12-14) was approved and followed a set of guidelines from IACUC. All animal procedures and studies were approved by the Cold Spring Harbor Laboratory Animal Use Committee in accordance to IACUC procedures. Animal experiments were performed with oversight of the Cold Spring Harbor Laboratory Animal Core Facility.

Note that full information on the approval of the study protocol must also be provided in the manuscript.

Flow Cytometry

Plots

Confirm that:

☒ The axis labels state the marker and fluorochrome used (e.g. CD4-FITC).

☒ The axis scales are clearly visible. Include numbers along axes only for bottom left plot of group (a 'group' is an analysis of identical markers).

☐ All plots are contour plots with outliers or pseudocolor plots.

☒ A numerical value for number of cells or percentage (with statistics) is provided.

Methodology

Sample preparation	4T1 cells were harvested and resuspended in basic sorting buffer (final conc. 2% FBS, 1 mM EDTA and 25mM HEPES pH 7.0)
Instrument	Becton Dickinson FACSAria II SORP 5 laser system/ Becton Dickinson LSR II
Software	Becton Dickinson FACSDiva 8.0.1
Cell population abundance	A sort gate was drawn around the double positive population and sorted into a 96-well plate using Single Cell Precision mode. Cells were cultured and examined by Genomic DNA PCR and Sanger Sequencing as well as qRT-PCR.
Gating strategy	A dot plot of FSC-A vs SSC-A was used to gate out debris (P1). A second dot plot of SSC-H vs SSC-W was gated off of P1 and used to gate out aggregates (P2). A third plot of FSC-H vs FSC-W was gated off of P2 and used to gate the Single cell population (P3). Lastly, another dot plot of GFP-A vs mCherry-A was gated off the single cell (P3) population for single cell sorting.<br>Figures for exemplifying the gating strategy are in the Source Data > Figure 2a

☒ Tick this box to confirm that a figure exemplifying the gating strategy is provided in the Supplementary Information.
