# Supplementary material for: MaTAR25 lncRNA regulates the Tensin1 gene to impact breast cancer progression
Source: Nat Commun. 2020 Dec 22;11:6438. doi: 10.1038/s41467-020-20207-y (PMC7755919; doi:10.1038/s41467-020-20207-y)
Supplement: Supplementary file 3 — Description of Additional Supplementary Files [file 41467_2020_20207_MOESM3_ESM.pdf]

Description of Additional Supplementary Files

File Name: Supplementary Data 1
Description: *MaTAR25* KO RNAseq differentiation expression genes list

File Name: Supplementary Data 2
Description: 4T1 cells *MaTAR25* ChIRP-seq targeting regions list

File Name: Supplementary Data 3
Description: cNeu (MMTV-NeuNDL) cells *MaTAR25* ChIRP-seq targeting regions list

File Name: Supplementary Data 4
Description: 4T1 cells *MaTAR25* ChIRP-seq and KO RNAseq overlapping genes

File Name: Supplementary Data 5
Description: List of antibodies used in this study

File Name: Supplementary Data 6
Description: List of reagents used in this study

File Name: Supplementary Data 7
Description: List of assays and resources used in this study

File Name: Supplementary Data 8
Description: List of oligonucleotides used in this study

File Name: Supplementary Data 9
Description: 4T1 cells *MaTAR25* oligos pulldown_MS_iTRAQ protein list

File Name: Supplementary Data 10
Description: FACS figures for gating/sorting 4T1 CRISPR/Cas9 positive cells

File Name: Supplementary Movie 1
Description: Video of 4T1 migration assay (4T1 control1)

File Name: Supplementary Movie 2
Description: Video of 4T1 migration assay (4T1 *MaTAR25* KO2)
